# Selective Cleavage of Lignin Model Compounds via a Reverse Biosynthesis Mechanism

**DOI:** 10.1021/acs.orglett.3c01416

**Published:** 2023-06-09

**Authors:** Sang Mi Suh, Subramanian Jambu, Mason T. Chin, Tianning Diao

**Affiliations:** Department of Chemistry, New York University, 100 Washington Square East, New York, New York 10003, United States

## Abstract

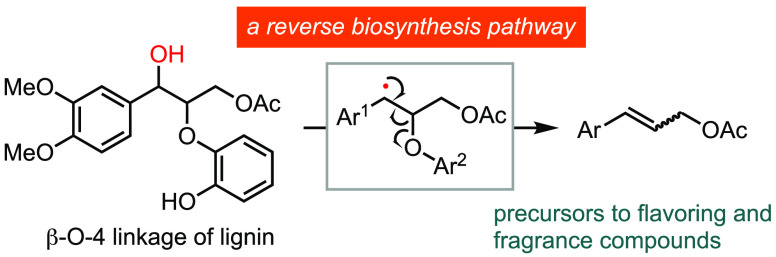

Selective depolymerization of lignin remains a significant challenge in biomass conversion. The biosynthesis of lignin involves the polymerization of monolignol building blocks through oxidative radical coupling reactions. A strategy for lignin degradation leverages photoredox deoxygenative radical formation to trigger reverse biosynthesis, which cleaves model compounds of the β-O-4 and β-5-β-O-4 linkages to produce monolignols, precursors to flavoring compounds. This mild method preserves important oxygen functionality and serves as a platform for achieving selective lignin depolymerization.

Lignocellulose biomass is a valuable renewable source that can be utilized for fuels, chemicals, and energy. The lignin component, which comprises 15–30% of lignocellulose biomass by weight, is not utilized to its full potential, with over 40 million tons discarded and incinerated each year.^1,2^ Lignin is synthesized naturally through the oxidation of phenylpropanoid monomers (monolignols) to phenolic radicals, **1**, which then dimerize to form C–O and C–C linkages (Scheme 1A).^3,4^ The different regioselectivity of radical dimerization gives rise to various motifs, including the β-O-4, β-5, and β–β linkages, which vary in composition among different plants. The low reactivity of the ether C–O bonds of these various linkages, as well as the irregular structure of lignin, imposes a significant challenge for selectively converting lignin into functional products under mild conditions.^5−7^

**Scheme 1:**
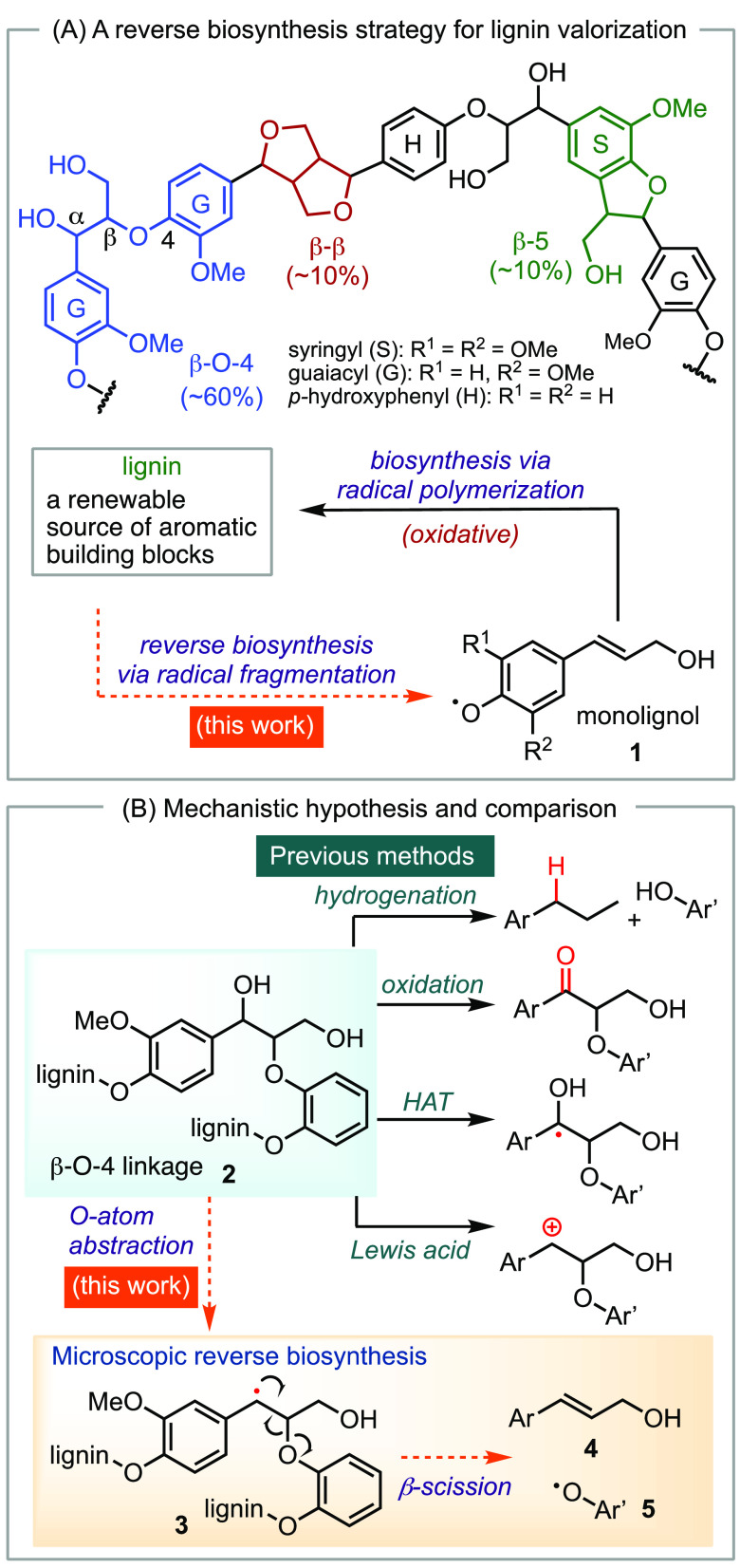
Biosynthesis of Lignin and Strategies for Depolymerization

While reductive catalytic fractionation (RCF) based on hydrogenation has achieved high conversion and selectivity,^8−10^ the process results in the loss of useful chemical functionalities of lignin through intensive hydrogenation under elevated temperature and high-pressure hydrogen gas (Scheme 1B). Alternative methods include oxidation of the α-hydroxyl group of the β-O-4 linkage **2**,^11−13^ hydrogenation of aryl ether or biaryl linkages,^14^ hydrogen-atom abstraction at the β-O-4 linkage **2**,^15−19^ and formation of carbocations with Lewis acids at the β-O-4 linkage **2**.^20^ These methods often result in a mixture of products that are difficult to purify and isolate despite high overall yields. Additionally, some of these mechanisms proceed through highly reactive intermediates, which results in undesirable pathways and diminished yields.^20^ Therefore, exploring other mechanistic modes for cleaving the β-O-4 linkage could provide valuable insight into developing lignin depolymerization methods that are selective for certain products.

Inspired by the biosynthesis of lignin, we report herein a depolymerization strategy based on a reverse biosynthesis pathway (Scheme 1A). This pathway involves a distinct mechanism from previous methods for lignin degradation.^21^ We hypothesize that an oxygen-atom abstraction can occur on the benzylic hydroxyl group of the β-O-4 linkage **2**, leading to the formation of a benzyl radical **3** (Scheme 1B), which is similar to the intermediates formed in the biosynthesis of lignin. The benzyl radical **3** can undergo β-scission of the adjacent C–O bond, which is a microscopic reverse step in the biosynthesis of lignin to afford monolignol **4** as the major product. The phenoxy radical **5** can propagate and cause further fragmentation or undergo chain termination via electron-transfer. We have previously demonstrated the effectiveness of this reverse-biosynthesis approach by applying the Nugent–RajanBabu reagent, Cp_2_Ti(III)Cl,^22,23^ to initiate the oxygen-atom abstraction.^24^ While the reaction showed high selectivity, the use of reductive conditions resulted in the reduction of the allylic alcohol of **4** into allyl groups. In this study, we prevent over-reduction by applying photoredox conditions to initiate deoxygenative radical formation. The resulting monolignol products are important precursors to flavoring and fragrance compounds.

In light of recent developments in photoredox oxygen-atom abstraction conditions,^25−28^ we report our investigation with two such conditions utilizing redox auxiliaries, dihydropyridine carboxylic acid (DHP-CO_2_H)^27^ and oxalyl chloride (COCl)_2_.^25^ Deoxygenative radical formation at the α-position of the β-O-4 linkage would initiate a reverse biosynthesis sequence and undergo fragmentation (Scheme 1B). Compared to titanium-catalyzed lignin degradation,^24^ these photoredox conditions offer high yields of a monolignol product with retained alcohol functionality. Additionally, we report an optimized synthesis of a β-5-β-O-4 lignin model substrate via an electro-oxidative [3 + 2] cycloaddition to forge the benzofuran core as a key step, which enabled the assessment of the reverse biosynthetic degradation of the β-O-4 linkage in the presence of other linkages.

We first tested the reactivity of DHP-CO_2_H as a redox auxiliary for facilitating deoxygenative radical formation and degradation of the β-O-4 model substrate **6** (Scheme 2). The condensation of DHP-CO_2_H with **6** afforded the corresponding DHP-ester **7** in 74% yield under our previous conditions.^27^ Upon irradiation of **7** with 467 nm light in the presence of photocatalyst [Ir[dF(CF_3_)ppy]_2_(dtbpy)PF_6_**10**, **7** underwent fragmentation to generate phenol **8** in 56% yield and 3,4-dimethoxycinnamyl acetate **9** in 33% yield as a mixture of the *E* and *Z* diastereomers. The ratio of these diastereomers is roughly consistent with the d.r. of the starting material, suggesting that the benzylic radical has a short lifetime and that β-elimination occurs rapidly before the conformation of the molecule equilibrates to the thermodynamically stable isomer.

**Scheme 2:**
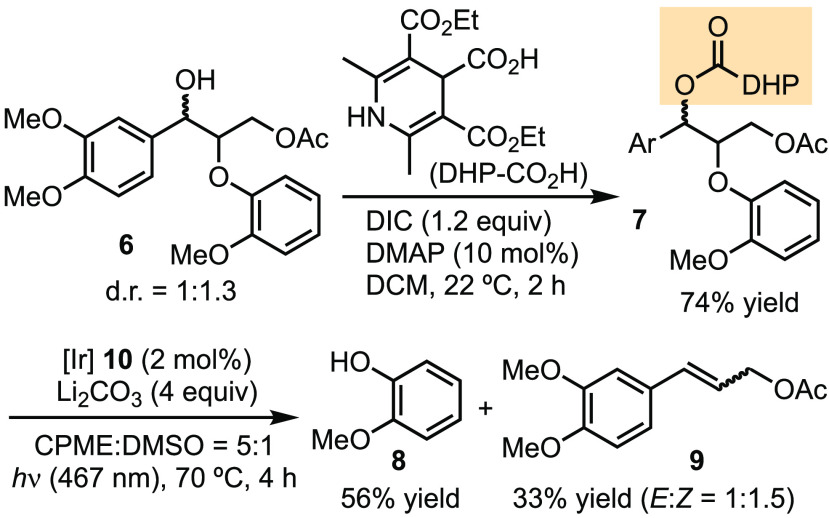
DHP-Mediated Deoxygenative Degradation of β-O-4 Model Substrate **6**

Subsequently, we investigated the use of oxalate ester as an auxiliary to activate **6**. Protection of **6** with (COCl)_2_ afforded **11** in 70% isolated yield (Table 1). We tested photoredox conditions that had previously been developed for the deoxygenative fragmentation of oxalate (Table 1).^25^ Upon exposure to these conditions, oxalic acid **11** underwent immediate fragmentation to give phenol **8** and a mixture of *E* and *Z* isomers of 3,4-dimethoxycinnamyl acetate **9**. The identity of base appears to be crucial to the yields (entries 1–11), with Li_2_CO_3_ being the most effective (entries 1–4). However, we did not observe a direct correlation between the yield/conversion and the p*K*_b_, possibly due to the interplay of multiple factors, including basicity, alkali ionic strength, and solubility. Other photocatalysts led to significantly lower conversion, and the absence of a photocatalyst resulted in nearly no conversion (entries 12–16).

**Table 1:**
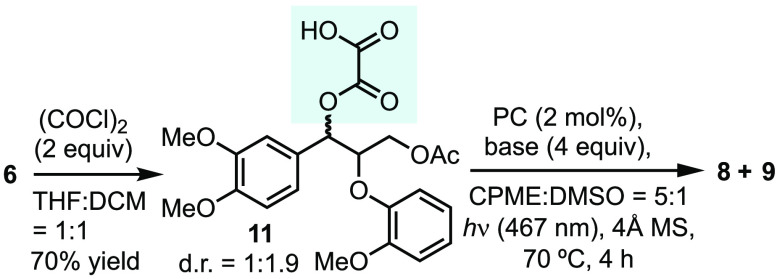
Degradation of β-O-4 Model Substrate **6** Initiated by Photoredox Fragmentation of Oxalate Ester^a^

entry	PC	base	% yield of **8**	% yield of **9** (*E*:*Z*)
**1**	**[Ir] 10**	**Li**_**2**_**CO**_**3**_	**89**	**88** (1:1.8)
**2**^b^	**[Ir] 10**	**Li**_**2**_**CO**_**3**_	**82**	**87** (1:1.9)
3^c^	[Ir] **10**	Li_2_CO_3_	57	81 (1:1.5)
4^d^	[Ir] **10**	Li_2_CO_3_	79	82 (1:1.8)
5	[Ir] **10**	Na_2_CO_3_	89	75 (1:1.9)
6	[Ir] **10**	K_2_CO_3_	85	68 (1:1.7)
7	[Ir] **10**	Cs_2_CO_3_	27	27 (1:2.4)
8	[Ir] **10**	NaHCO_3_	81	69 (1:1.6)
7	[Ir] **10**	Na_2_HPO_4_	62	56 (1:1.8)
9	[Ir] **10**	K_2_HPO_4_	64	57 (1:1.7)
10	[Ir] **10**	Na_3_PO_4_	87	76 (1:1.5)
11	[Ir] **10**	none	89	12
12	[Ir(ppy)_3_]	Li_2_CO_3_	3	trace
13	4CzIPN	Li_2_CO_3_	33	29
14	Mes-Acr-Me^+^	Li_2_CO_3_	4	trace
15	[Ru(bpy)_3_]^2+^	Li_2_CO_3_	3	trace
16	none	Li_2_CO_3_	5	trace

a Reaction conditions: [**11**] = 0.25 M (25 mg), photocatalyst (2 mol %), base (4 equiv), 4 Å MS = 25 mg, blue LED 467 nm (Kessil lamp), CPME = cyclopentyl methyl ether. Yields were determined by GC against mesitylene as the internal standard.

b Isolated yield with 100 mg substrate.

c **11** was not isolated and was directly subjected to photoredox degradation.

d In the absence of 4 Å MS.
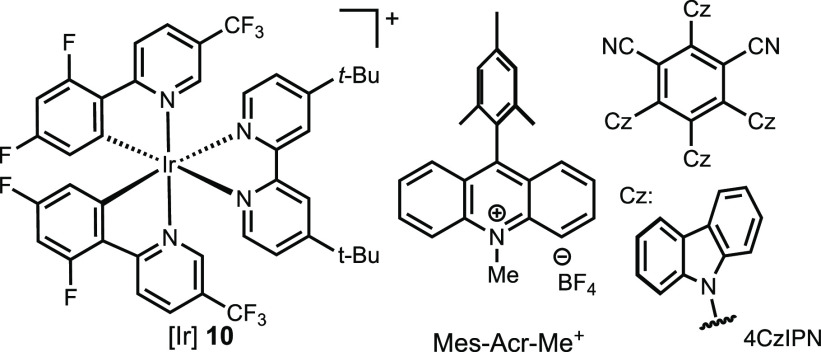

The addition of 4 Å molecular sieves (MS) provided a beneficial effect, possibly due to their capability of absorbing CO_2_ generated from the reaction, which promoted decarboxylation (entry 4). We also tested a one-pot process by forming the oxalate ester, followed by subjecting the crude mixture to photoredox degradation without column purification, resulting in comparable yields of **8** and **9** (entry 3).

The synthesis of lignin model substrates with various linkages is critical for evaluating lignin depolymerization conditions and probing mechanisms.^29^ While the synthesis of the β-O-4 linkage has been extensively practiced, analogous studies with the β-5 linkage is underdeveloped.^24^ Previous syntheses of this linkage suffers from low yield of a critical [3 + 2] cycloaddition step under chemical oxidative conditions (**13** → **14**, Scheme 3).^30^ We optimized the synthesis of a β-5-β-O-4 model compound applying recently reported electrocatalytic [3 + 2] cycloaddition conditions.^31^ Conducting the cycloaddition of **13** with *p*-methoxy-phenol under electrooxidation conditions resulted in the β-5 motif **14** in 87% yield. The ^3^*J*_*H-2*/*H-3*_ coupling constant of 8.0 Hz allowed us to assign the stereochemistry as *trans* based on previous reports.^32^ Subsequent functional group manipulation and condensation constructed the β-O-4 moiety, generating β-5-β-O-4 model compound **19**. Installation of the oxalate auxiliary furnished **20** in synthetically useful yield, enabling subsequent reactivity studies. Applying the optimized photoredox conditions identified in the study of **11** (entry 1, Table 1) led to the cleavage of **20** to generate **9** in 34% yield as a mixture of the *E*/*Z* isomers and phenol derivative **21** in 28% yield. We attribute the lower yield to an inefficient photoredox activation of oxalate in a larger molecule.

**Scheme 3:**
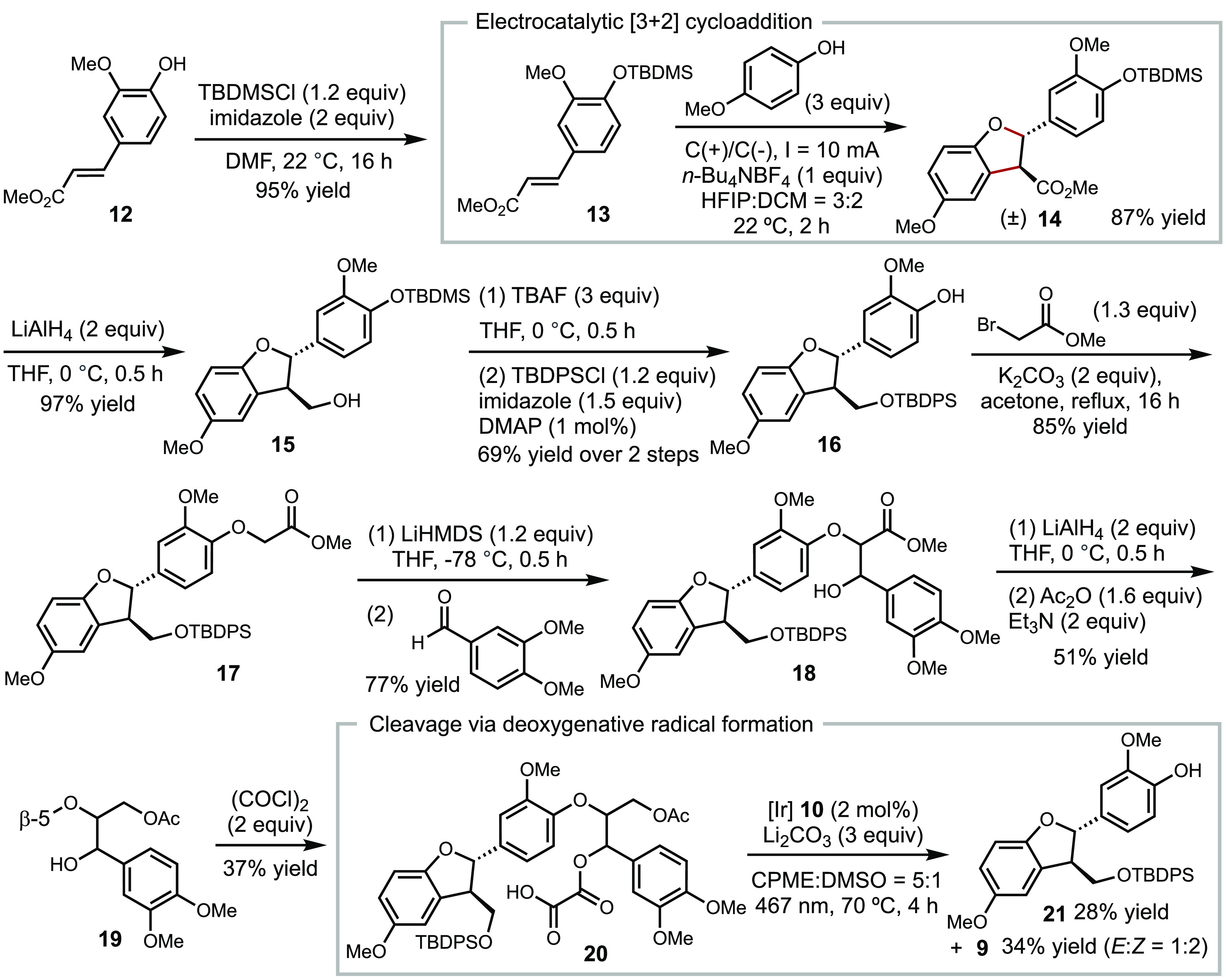
Optimized Synthesis of β-5-β-O-4 Model Compound **19** and Its Cleavage via Deoxygenative Radical Formation

In summary, we have utilized photoredox conditions to selectively degrade lignin model substrates containing the β-O-4 and β-5-β-O-4 linkages. Deoxygenative radical formation at the α-position of the β-O-4 linkage triggers a reverse biosynthetic pathway, leading to the degradation of the β-O-4 linkage into monolignol precursors via subsequent β-scission. This mechanism has not been previously explored among lignin degradation strategies. Under these conditions, the degradation of a β-O-4 model substrate was successful to generate phenol and 3,4-dimethoxycinnamyl acetate in high yields, while the dimeric β-5-β-O-4 model substrate proceeded to afford the degradation products in a lower yield.

## Data Availability

Data underlying this study are available in the published article and its Supporting Information.

## Supporting Information Available

Experimental procedures, additional data, and characterization data of new compounds (PDF)
