# Supplementary material for: Selective Cleavage of Lignin Model Compounds via a Reverse Biosynthesis Mechanism
Source: Org Lett. 2023 Jun 9;25(26):4792–6. doi: 10.1021/acs.orglett.3c01416 (PMC10334464; doi:10.1021/acs.orglett.3c01416)
Supplement: Supplementary file 1 — ol3c01416_si_001.pdf [file ol3c01416_si_001.pdf]

Selective Cleavage of Lignin Model Compounds via a Reverse Biosynthesis Mechanism

Sang Mi Suh, Subramanian Jambu, Mason T. Chin, and Tianning Diao*

Department of Chemistry, New York University, New York 10003, United States

*E-mail: diao@nyu.edu

Table of Contents

1. General Considerations……………………………………………….….…………….S2
2. Preparation of β-O-4 Model Compound **11**…..…………………….………………….S3
3. Additional Optimization Data..…………………..……………………….………...S8
4. Procedure for the photocatalytic cleavage of the β-O-4 on 100 mg scale……..…..….S10
5. Preparation of β-5-β-O-4 model substrate **20** ……………………………….……….S11
6. NMR Spectra………………………………….…………………….…….….……….S18
7. References…………………………………………………………….………...S31

1. General Considerations

Equipment and Methods

All air- and moisture- sensitive manipulations were performed either in a nitrogen-filled glove box or using Schlenk technique. ^{1}H, and ^{13}C, spectra were recorded on Bruker 400 MHz Avance spectrometer. Chemical shifts of the ^{1}H resonances are reported in ppm relative to tetramethylsilane, with the residual solvent resonance ($CDCl_3$, δ = 7.26 ppm, acetone-d_6, δ = 2.05 ppm, and DMSO-d_6, δ = 2.50 ppm) as the internal reference. Spectra are reported as the following: chemical shift (δ ppm), multiplicity (s = singlet, b s = broad singlet, d = doublet, t = triplet, q = quartet, m = multiplet), coupling constant (Hz), and integration. Chemical shifts of the ^{13}C were reported in ppm relative to tetramethylsilane with the solvent resonance used as the internal reference ($CDCl_3$, δ = 77.2 ppm DMSO-d_6, δ = 39.52 ppm). GC-MS data was obtained using a Shimadzu GCMS-TQ8040 with a Shimadzu SH-Rxi-5Sil MS column (L 30 m, ID 0.25 mm, DF 0.25 µm). GC data was obtained using a Shimadzu GC-2010 Plus with a Restek Rxi-5ms column (L 15 m, ID 0.25 mm, DF 0.25 µm). High resolution mass spectra (HRMS) were collected on an Agilent 6224 TOF LC/MS. Reactions were monitored by thin-layer chromatography (TLC) on Merck TLC silica gel 60 F254 plates and compounds were visualized by UV light (254 nm) or staining with iodine and $KMnO_4$.

Reagents and Solvents

All commercially available compounds were purchased and used as received, unless otherwise noted. Solid substrates and reductants were dried overnight under reduced pressure (~20 mTorr) before being brought into a nitrogen filled glove box. [(Ir[dF(CF_3)ppy]$_2$(dtbpy)) PF_6] were prepared according to literature procedures.[1] DMA (DMA = dimethylacetamide), DMF (DMF = Dimethylformamide), THP (THP = Tetrahydropyran), and CPME (CPME = Cyclopentyl methyl ether) were stored over molecular sieves for several weeks and deoxygenated before use. Dimethyl sulfoxide (DMSO) was dried over CaH_2, distilled, and degassed before use. 4Å molecular sieves (MS) were activated by heating under evacuation and stored in the glove box.

Photochemical Equipment and Setup:

The light source used for the photoredox reactions: Kessil PR160L 467 nm. (For more details about the lamp, please go to https://www.kessil.com/products/science_PR160L.php to check). The sealed vial was positioned on a stir plate in an oil bath approximately 4.5 cm away from two lamps.

2. Preparation of β-O-4 Model Compound **11**

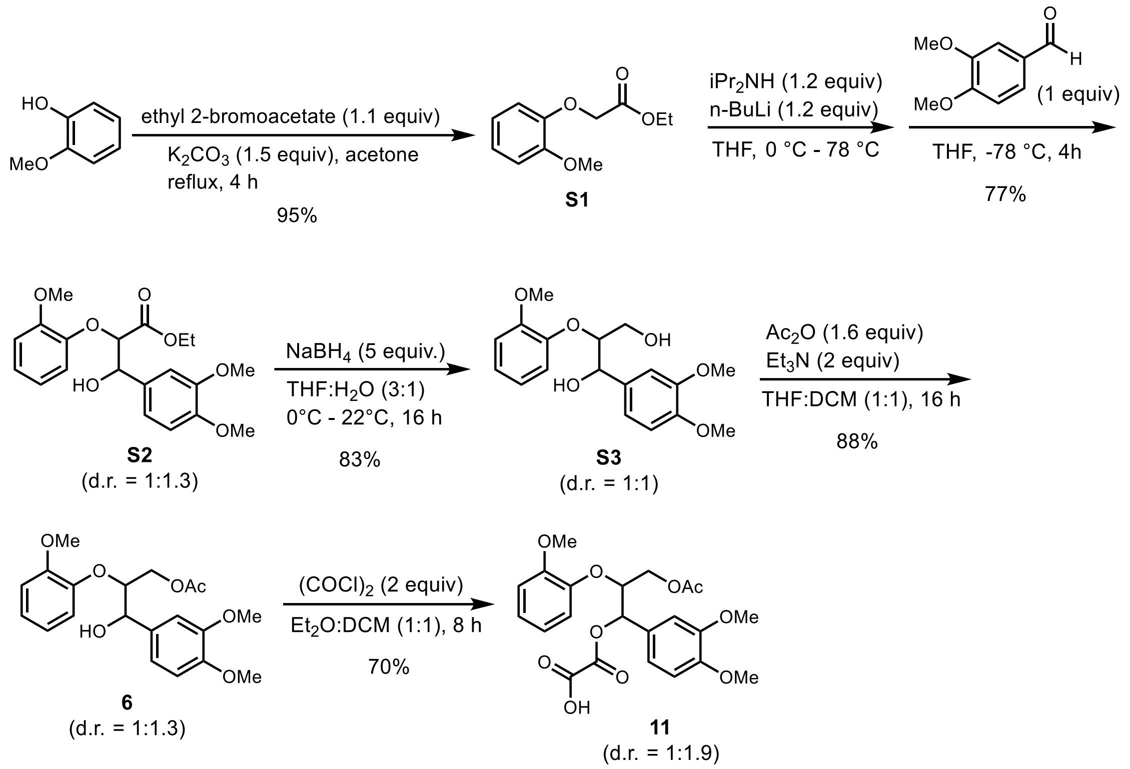

Scheme S1: Synthesis of β-O-4 Model Lignin Compound.

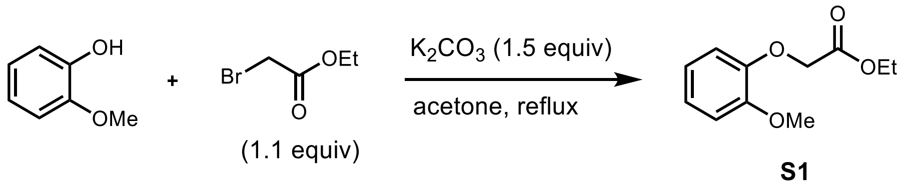

S1

Ethyl 2-(2-methoxyphenoxy)acetate (S1). A flame-dried 250 mL flask equipped with a stir bar was charged with guaiacol (7.9 mL, 70.6 mmol, 1 equiv), methyl bromoacetate (13.0g, 77.7 mmol, 1.1 equiv), K_2CO_3 (14.6 g, 105.9 mmol, 1.5 equiv), and acetone (230 mL, 0.30 M). After purging with N_2, the reaction was heated to reflux in an oil bath and stirred at reflux for 4 h. The resulting reaction mixture was cooled to room temperature and filtered through a pad of Celite. The filtrate was concentrated *in vacuo* to afford **S1** as a colorless liquid (14.1 g, 67.1 mmol) in 95% yield. The spectroscopic data of diol **S1** are consistent with literature data.[2] ^{1}H NMR (400 MHz, $CDCl_3$) δ 7.00 -6.94 (m, 1H), 6.93 -6.82 (m, 3H), 4.67 (s, 2H), 4.25 (q, J = 7.1 Hz, 2H), 3.87 (s, 3H), 1.28 (t, J = 7.1 Hz, 3H). ^{13}C NMR (101 MHz, $CDCl_3$) δ 169.2, 149.8, 147.4, 122.6, 120.8, 114.6, 112.2, 66.7, 61.3, 56.0, 14.3.

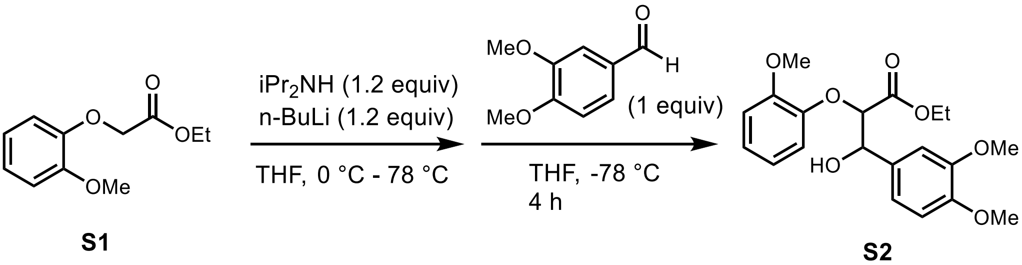

S1 **S2**

Ethyl 3-(3,4-dimethoxyphenyl)-3-hydroxy-2-(2-methoxyphenoxy)propanoate (S2). A flame dried 250 mL three-neck round bottom flask equipped with a stir bar was evacuated and filled with N_2 using Schlenk technique. After the addition of diisopropylamine (10.3 mL, 73.3 mmol, 1.1 equiv) and THF (110 mL, 0.6 M) *via* syringe under positive N_2 pressure, the reaction was cooled to 0°C in an ice bath. 1.8 M *n*-BuLi (35 mL, 73.3 mmol, 1.1 equiv) was added dropwise and the mixture stirred for 15 min at 0 °C. The reaction was then cooled to -78 °C in an acetone/dry ice bath and charged with a solution of **S1** (14.0 g, 66.6 mmol, 1.0 equiv) in 35 mL THF over 15 min using a syringe pump. The reaction mixture stirred for 30 min at -78 °C, after which a solution of 3,4-dimethoxybenzaldehyde (11.1 g, 66.6 mmol, 1 equiv) in 35 mL THF was added dropwise. After stirring for 4 h at -78 °C, followed by quenching with a solution of saturated aqueous NH_4Cl and warming up to room temperature to give a two-layered mixture. The organic layer was separated and the aqueous phase was extracted with EtOAc (3 x 75 mL). The combined organic layers were dried over Na_2SO_4, filtered and concentrated *in vacuo* to afford a yellow liquid. The crude residue was purified by flash column chromatography (1:1 hexane:EtOAc) to afford **S2** as a colorless liquid (19.3 g, 51.3 mmol) in 77% yield. The spectroscopic data of diol **S2** are consistent with literature data.[3] Ratio of diastereomers (minor to major): 1:1.3, determined by ^{1}H NMR spectroscopy. Spectroscopic data are reported for the mixture of isomers. ^{1}H NMR (400 MHz, $CDCl_3$) δ 7.08-6.80 (m, 7H), 5.15 and 5.06 (t, *J* = 5.4 Hz and dd, *J* = 7.0, 3.1 Hz, 1H), 4.74 and 4.49 (d, *J* = 5.0 Hz and d, *J* = 7.0 Hz, 1H), 4.15 – 3.99 (m, 2H), 3.91 (ddd, J = 6.9, 5.1, 3.0 Hz, 1.6H), 3.88 – 3.80 (m, 9H), 1.15 and 1.07 (t, *J* = 7.1 Hz and t, *J* = 7.1 Hz, 3H). ^{13}C NMR (101 MHz, $CDCl_3$) δ 171.2, 169.5, 169.4, 151.4, 150.5, 150.3, 150.2, 149.3, 149.1, 149.0, 148.8, 148.7, 148.5, 147.32, 147.26, 143.1, 133.7, 131.9, 130.7, 127.9, 123.8, 123.7, 123.6, 123.2, 121.12, 121.11, 119.6, 119.4, 119.3, 118.5, 117.9, 117.7, 112.34, 112.30, 111.1, 110.8, 110.7, 110.5, 110.2, 110.1, 85.2, 83.8, 74.8, 73.9, 65.2, 61.2, 60.4, 56.0, 56.0, 55.92, 55.90, 55.87, 55.85, 55.8, 14.2, 14.1, 14.0.

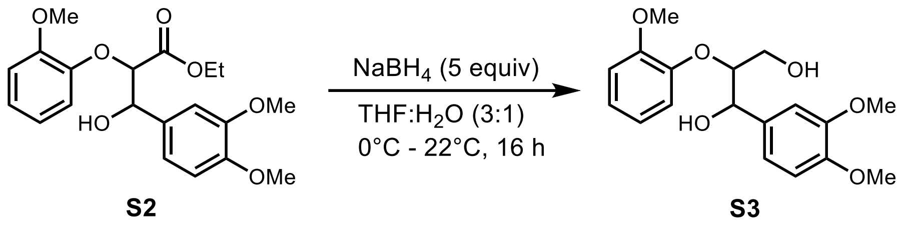

1-(3,4-Dimethoxyphenyl)-2-(2-methoxyphenoxy)-1,3-propanediol (S3). In a flame dried 250 mL flask equipped with a stir bar, **S2** (12.8 g, 34.0 mmol, 1.0 equiv) was dissolved in a 3:1 mixture of THF:H_2O (240 mL, 0.14 M). Sodium borohydride (6.4 g, 170 mmol, 5.0 equiv) was added in two-portions over 30 minutes at 0°C in an ice bath and the reaction mixture stirred overnight at room temperature. The resulting mixture was diluted with 160 mL deionized H_2O and 120 mL EtOAc, followed by phase separation. The aqueous phase was extracted with EtOAc (3 x 80 mL) and the organic layers were combined, dried over Na_2SO_4, filtered, and concentrated. The crude residue was purified by flash column chromatography (1:2 hexane:EtOAc) to afford **S3** as a colorless liquid (9.5 g, 31 mmol) in 83% yield. The spectroscopic data of diol **S2** are consistent with literature data.[4] Ratio of diastereomers (minor to major): 1:1, determined by ^{1}H NMR spectroscopy. Spectroscopic data are reported for the mixture of isomers. ^{1}H NMR (400 MHz, $CDCl_3$) δ 7.14 – 6.80 (m, 7H), 4.99 and 4.97 (d, *J* = 5.2 Hz and d, *J* = 2.1 Hz, 1H), 4.16 and 4.02 (ddd, *J* = 5.9, 4.8, 3.4 Hz and dt, *J* = 8.0, 3.5 Hz, 1H), 3.90-3.83 (m, 9H), 3.70-3.59 (m, 1H), 3.48 (dd, *J* = 12.5, 3.9 Hz, 1H), 2.81 (s, 1H). ^{13}C NMR (101 MHz, $CDCl_3$) δ 151.5, 151.2, 149.1, 149.0, 148.9, 148.5, 147.7, 147.0, 132.7, 132.3, 124.2, 124.1, 121.7, 121.6, 120.9, 120.7, 119.6, 119.4, 118.5, 112.2, 111.08, 111.06, 111.0, 110.5, 109.9, 109.3, 89.3, 87.2, 73.9, 72.8, 61.1, 60.8, 55.94, 55.90.

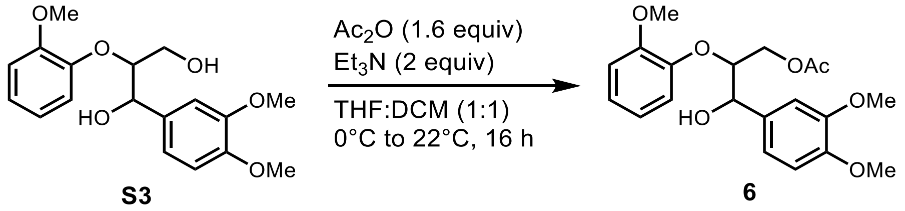

3-(3,4-Dimethoxyphenyl)-3-hydroxy-2-(2-methoxyphenoxy)propyl acetate (6). In a flame dried 250 mL flask equipped with a stir bar, **S3** (5.8 g, 17.4 mmol, 1.0 equiv) was dissolved in a 1:1 mixture of THF:DCM (170 mL, 0.1 M). Triethyl amine (4.8 mL, 34.8 mmol, 2 equiv) was added, and the reaction was cooled to 0 °C in an ice bath. Acetic anhydride (2.6 mL, 27.8 mmol, 1.6 equiv) was added over three portions and the reaction mixture was stirred overnight at room temperature. The resulting mixture was diluted with 80 mL of deionized water and 60

mL of DCM, followed by phase separation. The aqueous phase was extracted with DCM (3 x 80 mL) and the organic layers were combined, dried over Na_2SO_4, filtered, and concentrated. The crude residue was purified by flash chromatography (1:1 hexane:EtOAc) to afford **6** as a colorless liquid (5.8 g, 15 mmol) in 88% yield. Ratio of diastereomers (minor to major): 1:1.3, determined by ^{1}H NMR spectroscopy. Spectroscopic data are reported for the mixture of isomers. ^{1}H NMR (400 MHz, $CDCl_3$) δ 7.17 – 6.80 (m, 13H), 4.90 (d, *J* = 3.9 Hz, 1H), 4.87 (d, *J* = 8.2 Hz, 1H), 4.44 (dt, *J* = 7.2, 3.5 Hz, 1H), 4.38 (dd, *J* = 11.7, 7.5 Hz, 1H), 4.27 – 4.18 (m, 2H), 4.12 (dd, *J* = 11.7, 3.2 Hz, 1H), 4.06 – 3.99 (m, 1H), 3.93 – 3.85 (m, 17H), 2.04 (s, 2H), 2.00 (s, 3H).^{13}C NMR (101 MHz, $CDCl_3$) δ 171.1, 170.7, 151.7, 151.0, 149.3, 149.2, 149.1, 148.6, 148.2, 147.1, 131.9, 131.6, 124.31, 124.28, 124.2, 121.59, 121.56, 121.1, 120.9, 120.6, 119.93, 119.88, 118.6, 112.5, 112.32, 112.28, 111.2, 111.1, 109.9, 109.5, 86.3, 86.1, 84.6, 77.5, 77.1, 76.8, 74.4, 74.0, 72.1, 63.4, 63.0, 56.1, 56.03, 56.01, 55.99, 55.9, 21.3, 21.0, 20.9. HRMS: m/z (ESI) Calcd for $C_{20}H_{24}NaO_7$ $[M+Na]^+$:399.1420, found: 399.1442.

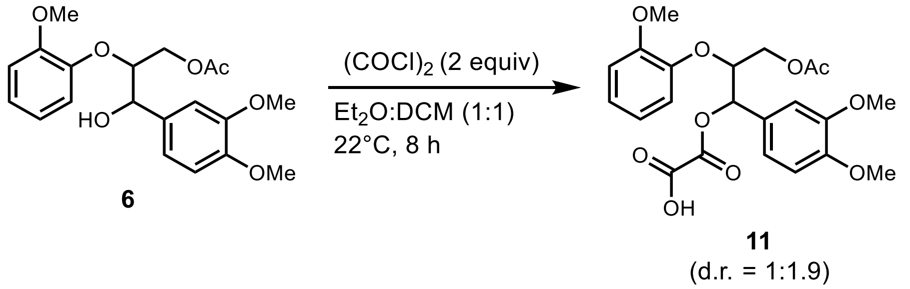

2-(3-Acetoxy-1-(3,4-dimethoxyphenyl)-2-(2-methoxyphenoxy)propoxy)-2-oxoacetic acid (11). In a flame dried 250 mL flask equipped with a stir bar, **6** (3.9 g, 10.4 mmol, 1 equiv) was dissolved in a 1:1 mixture of Et_2O:DCM (50 mL). Oxalyl chloride (1.8 mL, 20.8 mmol, 2 equiv) was added dropwise and the reaction mixture stirred at room temperature for 8 h. The resulting yellow oil was redissolved in Et_2O (50 mL) and carefully treated with deionized water (50 mL). The biphasic reaction mixture was stirred vigorously for 1 hour and the layers were separated. The aqueous phase was extracted with Et_2O (3 x 50 mL) and the combined ethereal extracts were washed with water (2 x 100 mL) and brine (100 mL), dried over Na_2SO_4 filtered, and concentrated. The crude residue was purified by flash chromatography (1:3 hexane:EtOAc) to afford **11** as a colorless liquid (3.2 g, 7.1 mmol) in 70% isolated yield. Ratio of diastereomers (minor to major): 1:1.9, determined by ^{1}H NMR spectroscopy. Spectroscopic data are reported for the mixture of isomers. ^{1}H NMR (400 MHz, acetone-d_6) δ 7.21-6.80 (m, 7H), 6.23 and 6.17 (d, J = 7.5 Hz and d, *J* = 5.1 Hz, 1H), 4.95-4.85 (m, 1H), 4.40, 4.26, and 4.01 (dd, *J* = 11.9, 5.9

Hz, ddd, J = 12.0, 3.9, 1.1 Hz, and dd, J = 12.0, 5.1 Hz, 2H), 3.86-3.77 (m, 9H), 1.99 and 1.96 (s, 3H). ^{13}C NMR (101 MHz, acetone-d_6) δ 170.8, 170.7, 159.0, 158.9, 158.6, 152.1, 152.0, 151.0, 150.7, 150.4, 150.3, 150.2, 149.1, 148.1, 128.8, 128.7, 124.2, 124.0, 123.6, 121.8, 121.7, 121.6, 121.3, 121.2, 121.1, 120.0, 119.82, 119.76, 119.7, 119.3, 119.1, 113.73, 113.68, 113.6, 112.5, 112.4, 112.33, 112.30, 112.23, 112.17, 111.8, 84.2, 83.3, 81.2, 80.9, 80.2, 78.6, 77.3, 75.7, 64.1, 63.9, 63.6, 63.0, 56.3, 56.2, 56.14, 56.10, 56.06, 56.0, 54.1, 49.7, 21.0, 20.62, 20.60. HRMS: m/z (ESI) Calcd for $C_{22}H_{24}NaO_{10}$ $[M+Na]^+$: 471.1262, found: 471.1273.

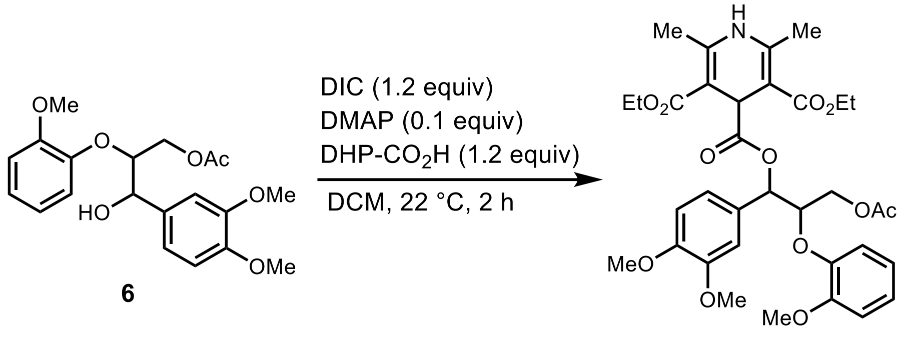

(d.r. = 1:1.4)

4-(3-acetoxy-1-(3,4-dimethoxyphenyl)-2-(2-methoxyphenoxy)propyl) 3,5-diethyl 2,6-dimethyl-1,4-dihydropyridine-3,4,5-tricarboxylate (7). In a flame dried 100 mL flask equipped with a stir bar, **6** (500 mg, 1.3 mmol, 1 equiv) was dissolved DCM (20 mL). DIC (0.25 mL, 1.6 mmol, 1.2 equiv), DMAP (16 mg, 0.13 mmol, 0.1 equiv), and 3,5-bis(ethoxycarbonyl)-2,6-dimethyl-1,4-dihydropyridine-4-carboxylic acid (474 mg, 1.6 mmol. 1.2 equiv) was added to the reaction mixture and stirred at room temperature for 2 h. Diethyl ether (10 mL) was added to the reaction mixture and the mixture was filtered through Celite. The filtrate was concentrated *in vacuo* and the crude residue was purified by flash column chromatography (95:5 DCM:MeOH) to afford **7** as a colorless oil (640 mg, 0.98 mmol) in 74% yield. Ratio of diastereomers (minor to major): 1:1.4, determined by ^{1}H NMR spectroscopy. Spectroscopic data are reported for the mixture of isomers. ^{1}H NMR (400 MHz, $CDCl_3$) δ 8.55 (d, J = 7.7 Hz, 1H), 7.55 – 7.49 (m, 1H), 7.16 – 6.69 (m, 7H), 5.13 (s, 1H), 4.67 – 4.51 (m, 2H), 4.44 – 3.93 (m, 4H), 3.91 – 3.62 (m, 9H), 2.33 – 2.14 (m, 6H), 1.99 (dd, J = 18.8, 13.1 Hz, 3H), 1.34 – 1.13 (m, 6H). ^{13}C NMR (101 MHz, $CDCl_3$) δ 175.2, 172.3, 171.0, 170.7, 168.4, 167.4, 167.3, 167.21, 167.16, 167.1, 153.6, 151.1, 150.9, 149.1, 149.0, 148.95, 148.90, 148.0, 147.3, 146.3, 146.2, 146.0, 145.94, 145.86, 129.6, 129.0, 124.2, 123.4, 123.0, 121.6, 121.14, 121.09, 120.6, 119.9, 119.7, 119.5, 119.4, 118.4, 112.7, 112.6, 112.3, 111.1, 110.9, 110.8, 110.6, 110.5, 109.9, 99.6, 98.6, 98.41, 98.37, 98.3, 86.3, 80.6, 80.0, 74.6, 74.4, 74.1, 63.5, 63.4,

63.0, 60.5, 60.20, 60.17, 60.13, 60.08, 56.02, 56.01, 55.93, 55.91, 47.9, 43.0, 41.0, 40.43, 40.38, 22.5, 20.92, 20.90, 20.8, 20.3, 19.6, 19.3, 19.21, 19.17, 14.6, 14.48, 14.46, 14.44, 14.38. HRMS: m/z (ESI) Calcd for $C_{34}H_{41}NNaO_{12}$ $[M+Na]^+$:678.2526, found: 678.2540.

3. Additional Optimization Data

General Procedure for the photocatalytic lignin depolymerization of **11**

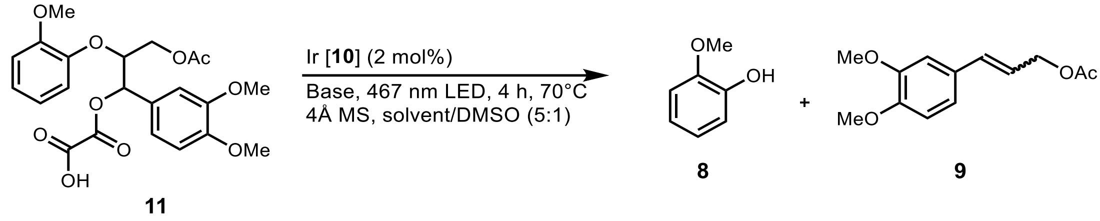

In an N_2-filled glove box, $[Ir[dF(CF_3)ppy]_2(dtbpy)]PF_6$ (2 mol %), base (4 equiv), and 4Å MS (25 mg), were added to a 4 mL vial equipped with a stir bar. The vial was sealed with a rubber septum and moved outside of the glovebox. The vial was then charged with CPME and a solution of **11** in DMSO, which was purged with N_2 prior to the addition. The reaction was stirred at 70 °C in an oil bath for 4 hours under blue LED light irradiation (467 nm, Kessil lamp). After the reaction, the mixture was quenched with saturated aqueous NH_4Cl (0.4 mL) and diluted with EtOAc (0.8 mL). The aqueous phase was extracted with EtOAc (2 x 0.8 mL) filtered through a pipette filled with a plug of Na_2SO_4, added 2.5 µL of mesitylene as an internal standard, and subjected to GC analysis.

Table S1. Effect of Solvents[a]

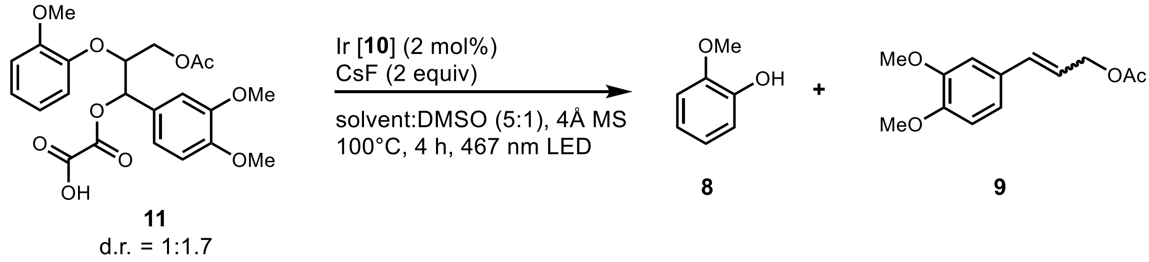

Entry	Solvent	% Yield of **8**	% Yield of **9** *(E:Z)*
1	DMF	10	Trace
2	DMA	11	13 (1:1.6)
3	THP	26	54 (1:1.4)
4	CPME	43	56 (1:1.4)
**5**	1,4-Dioxane	42	57 (1:1.3)

[a]GC yields were reported using mesitylene as an internal standard

Table S2. Effect of Reaction Time[a]

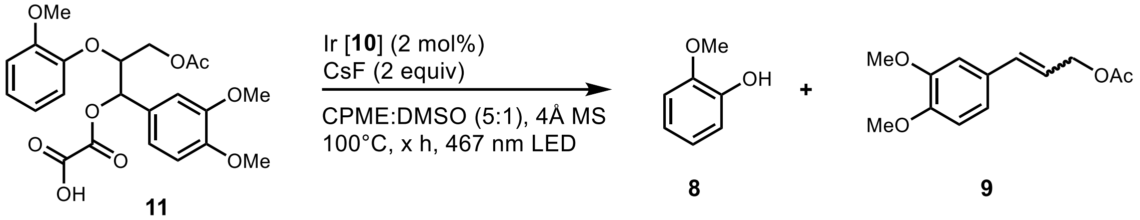

11
d.r. = 1:1.6
8
9

Entry	Time (h)	% Yield of **8**	% Yield of **9** *(E:Z)*
1	1	23	37 (1:1.6)
2	2	35	55 (1:1.3)
3	4	43	56 (1:1.4)
4	5	28	34 (1:1.4)
5	16	30	36 (1:1.3)

[a]GC yields were reported using mesitylene as an internal standard.

Table S3. Effect of Temperature[a]

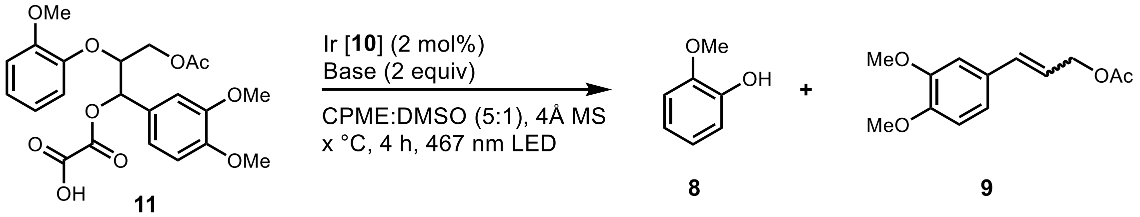

11
d.r. = 1:1.8
8
9

Entry	Base	Temperature	% Yield of **8**	% Yield of **9** *(E:Z)*
1	CsF	70 °C	43	56 (1:1.4)
2	Na_2HPO_4	40 °C	21	17 (1:1.4)
3	Na_2HPO_4	80 °C	28	43 (1:1.5)
4	Na_2HPO_4	90 °C	32	21 (1:1.6)

[a]GC yields were reported using mesitylene as an internal standard.

4. Procedure for the photocatalytic cleavage of the β-O-4 on 100 mg scale

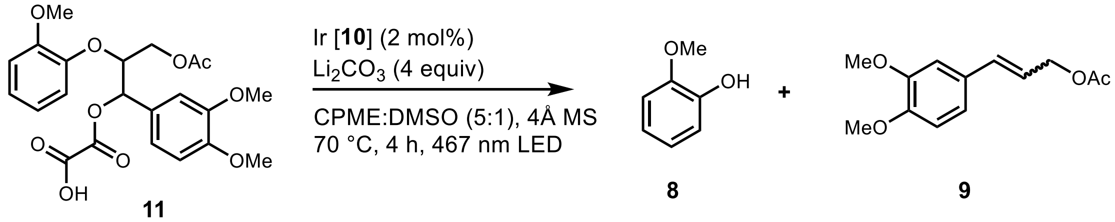

11 d.r = 1:1.7 **8** **9**

In a N_2-filled glove box, [(Ir[dF(CF_3)ppy]$_2$(dtbpy)) PF_6] (2 mol %), Li_2CO_3 (4 equiv), and 4Å MS (125 mg), were added 8 mL vial in glovebox with a stir bar. The vial was sealed with a rubber septum and moved outside of the glovebox. The vial was then charged with CPME and a solution of **11** in DMSO, which was purged with N_2 prior to the addition. The reaction was stirred at 70 °C in an oil bath for 4 hours under blue LED light irradiation (467 nm, Kessil lamp). After the reaction, the mixture was quenched with saturated aqueous NH_4Cl (2 mL) and diluted with EtOAc (3 mL). The aqueous phase was extracted with EtOAc (2 x 3 mL). The organic phase was dried over Na_2SO_4 filtered, and concentrated. The crude residue was purified by flash chromatography (95:5 hexane:EtOAc) to afford **8** as a colorless liquid (22.3 mg, 0.18 mmol) in 82% yield and **9** as a colorless liquid (45 mg, 0.19 mmol) in 87% yield. ^{1}H and ^{13}C NMR spectra are consistent with those reported in the literature.

2-Methoxyphenol[5] **8**

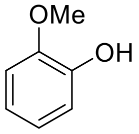

^{1}H NMR (400 MHz, $CDCl_3$) δ 6.98 – 6.82 (m, 4H), 5.63 (s, 1H), 3.89 (s, 3H). ^{13}C NMR (101 MHz, $CDCl_3$) δ 146.7, 145.8, 121.6, 120.3, 114.7, 110.8, 56.0

(*Z*)-3-(3,4-dimethoxyphenyl)allyl acetate/(*E*)-3-(3,4-dimethoxyphenyl)allyl acetate[6] **9**

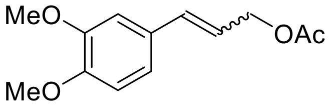

Ratio of diastereomers (minor to major): 1:1.9, determined by ^{1}H NMR spectroscopy. Spectroscopic data are reported for the mixture of isomers. ^{1}H NMR (400 MHz, $CDCl_3$) δ 6.95 – 6.90 (m, 1H), 6.88 – 6.75 (m, 2H), 6.59 and 5.73 (dd, *J* = 13.9, 4.4 Hz and dt, *J* = 11.8, 6.7 Hz, 1H), 4.84 and 4.70 (dd, *J* = 6.8, 1.6 Hz and d, *J* = 6.6 Hz, 1H), 3.92 – 3.83 (m, 6H), 2.08 (d, *J* = 4.0 Hz, 3H). ^{13}C NMR (101 MHz, $CDCl_3$) δ 171.0, 170.9, 149.3, 149.1, 148.8, 148.7,

134.4, 133.0, 129.3, 129.0, 124.4, 121.5, 121.2, 120.1, 112.0, 111.13, 111.06, 108.9, 77.5, 77.2, 76.8, 65.3, 61.6, 56.0, 55.92, 55.90, 21.1, 21.0.

5. Preparation of β-5-β-O-4 model substrate **20**

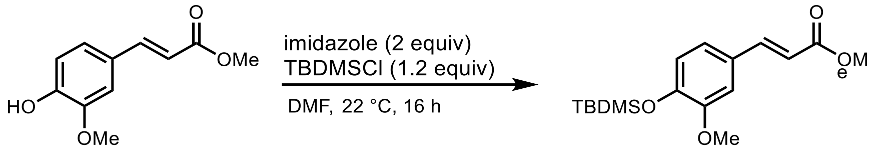

Methyl (*E*)-3-(4-((tert-butyldimethylsilyl)oxy)-3-methoxyphenyl)acrylate (13). In a flame dried 100 mL flask equipped with a stir bar, **12** (7.0 g, 33.61 mmol, 1.0 equiv) and *tert*-butyldimethylchlorosilane (6.1 g, 40.3 mmol, 1.2 equiv) was dissolved in DMF (20 mL). Imidazole (4.6 g, 67.2 mmol, 2 equiv) was added to the reaction mixture and the mixture stirred overnight at room temperature. The reaction solution was diluted with ethyl acetate (200 mL) and deionized water (100 mL). The organic layer was washed with saturated brine (100 mL) 1x. The organic layer was dried over anhydrous Na_2SO_4 and concentrated *in vacuo*. The crude residue was purified by flash column chromatography (9:1 hexane:EtOAc) to afford compound **13** as a white powder (10.3 g, 32 mmol) in 95% yield. ^{1}H NMR (400 MHz, $CDCl_3$) δ 7.62 (d, J = 15.9 Hz, 1H), 7.06 – 6.99 (m, 2H), 6.84 (d, J = 8.7 Hz, 1H), 6.30 (d, J = 15.9 Hz, 1H), 3.83 (s, 3H), 3.79 (s, 3H), 0.99 (s, 9H), 0.17 (s, 6H). ^{13}C NMR (101 MHz, $CDCl_3$) δ 167.9, 151.3, 147.7, 145.1, 128.4, 122.4, 121.2, 115.6, 111.0, 55.6, 51.7, 25.8, -4.5. HRMS: m/z (ESI) Calcd for $C_{17}H_{26}O_4Si$ $[M+H]^+$: 323.1673, found: 323.1651.

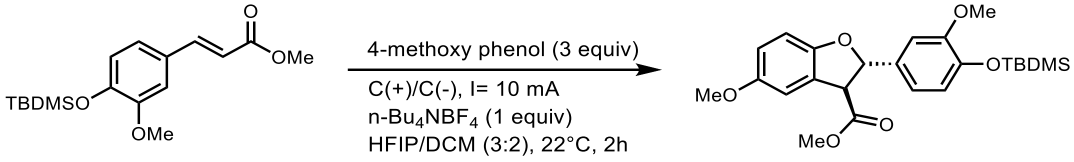

Methyl 2-(4-((tert-butyldimethylsilyl)oxy)-3-methoxyphenyl)-5-methoxy-2,3-dihydrobenzofuran-3-carboxylate (14). In a N_2-filled glove box, **13** (80 mg, 0.248 mmol, 1 equiv), *p*-methoxylphenol (92 mg, 0.74 mmol, 3 equiv), and n-Bu_4NBF_4 (82 mg, 0.25 mmol, 1 equiv) was added in a 10 mL ElectraSyn vial with stir bar. HFIP/CH_2Cl_2 (3:2, 8 mL) mixture was added to the reaction vial outside the box. The vial was equipped with graphite electrodes and stirred at a constant current of 10 mA at room temperature for 2 h under an N_2 atmosphere. The reaction mixture was concentrated *in vacuo* and the crude residue was purified by flash column chromatography (10:1 hexane:EtOAc) to give the **14** as an orange oil (96 mg, 0.22 mmol) in 87% yield. ^{1}H NMR (400 MHz, $CDCl_3$) δ 6.95 – 6.88 (m, 2H), 6.87 – 6.77 (m, 4H),

6.00 (d, J = 7.9 Hz, 1H), 4.27 (dt, J = 8.0, 0.9 Hz, 1H), 3.82 (s, 3H), 3.78 (s, 6H), 0.98 (s, 10H), 0.14 (s, 6H). ^{13}C NMR (101 MHz, $CDCl_3$) δ 171.4, 154.5, 153.5, 151.3, 145.3, 134.0, 125.0, 121.1, 118.5, 114.9, 111.2, 110.0, 109.9, 86.1, 56.2, 56.1, 55.6, 52.8, 25.8, -4.5. HRMS: m/z (ESI) Calcd for $C_{24}H_{33}O_6Si$ $[M+H]^+$: 445.2041, found: 445.2031.

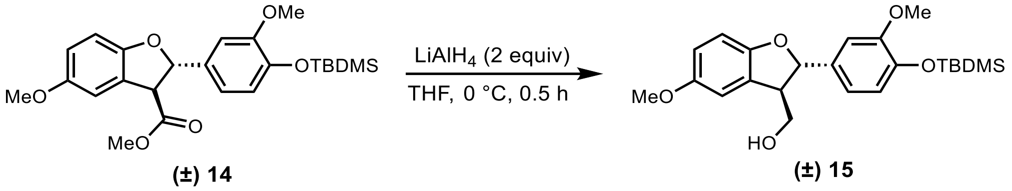

(2-(4-((Tert-butyldimethylsilyl)oxy)-3-methoxyphenyl)-5-methoxy-2,3-dihydrobenzofuran-3-yl)methanol (15). In a flame dried 100 mL flask equipped with a stir bar, $LiAlH_4$ (99 mg, 2.6 mmol, 2 equiv) was added to a solution of diethyl ether (25 mL). The reaction mixture was cooled to 0 °C in an ice bath and a solution of **14** (560 mg, 1.3 mmol, 1 equiv) in THF (5 mL) was added dropwise. After 30 minutes, the reaction solution was diluted with ethyl acetate (50 mL), followed by Fieser method workup. The organic layer was washed with saturated brine (50 mL), dried over Na_2SO_4 and concentrated *in vacuo*. The crude residue was purified by flash column chromatography (3:1 hexane:EtOAc) to afford **15** as a colorless oil (0.55 g, 1.3 mmol) in 97% yield. ^{1}H NMR (400 MHz, $CDCl_3$) δ 6.90 – 6.73 (m, 6H), 5.49 (d, J = 6.9 Hz, 1H), 3.98 (dd, J = 6.5, 4.5 Hz, 2H), 3.78 (d, J = 1.4 Hz, 6H), 3.61 – 3.53 (m, 1H), 0.98 (s, 9H), 0.14 (s, 6H). ^{13}C NMR (101 MHz, $CDCl_3$) δ 154.4, 154.2, 151.2, 145.0, 135.1, 127.9, 121.0, 118.5, 113.9, 110.8, 109.8, 109.6, 87.2, 64.2, 56.1, 55.6, 53.8, 25.8, 18.5, -4.53 HRMS: m/z (ESI) Calcd for $C_{23}H_{32}NaO_5Si$ $[M+Na]^+$: 439.1911, found: 439.1887.

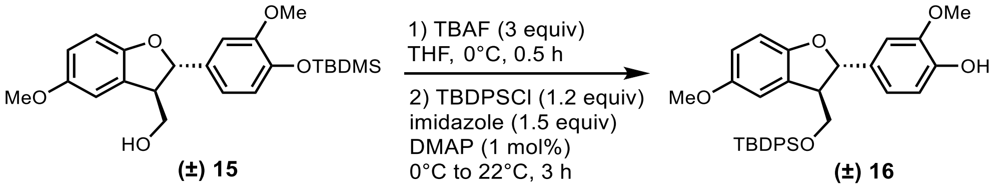

4-(3-(((tert-butyldiphenylsilyl)oxy)methyl)-5-methoxy-2,3-dihydrobenzofuran-2-yl)-2-methoxyphenol (16). In a flame dried 100 mL flask equipped with a stir bar, **15** (650 mg, 1.56 mmol, 1 equiv) was dissolved in THF (8 mL). A solution of tetrabutylammonium fluoride (1.5g, 4.68 mmol, 3 equiv) in THF (10 mL) was added dropwise to the reaction mixture. After 30 minutes, the reaction mixture was poured into a flask containing 30 mL of ice water and was stirred for 20 minutes. The aqueous phase was extracted with ethyl acetate (30mL x 3) and the combined organic phase was washed with saturated brine (30 mL) and dried over Na_2SO_4 and concentrated *in vacuo*. The crude residue was purified by flash column chromatography

(1:1 hexane:EtOAc) to afford 4-(3-(Hydroxymethyl)-5-methoxy-2,3-dihydrobenzofuran-2-yl)-2-methoxyphenol as a white solid (450 mg, 1.50 mmol).

In a flame dried 100 mL flask equipped with a stir bar, 4-(3-(Hydroxymethyl)-5-methoxy-2,3-dihydrobenzofuran-2-yl)-2-methoxyphenol (450 mg, 1.50 mmol, 1.0 equiv) was dissolved in dry DMF (3 mL). The reaction mixture was cooled to 0 °C in an ice bath and imidazole (153 mg, 2.25 mmol, 1.5 equiv), DMAP (2 mg, 0.015 mmol, 1 mol%) and *tert*-butyl(chloro)diphenylsilane (0.46 mL, 1.80 mmol, 1.2 equiv) was added slowly. The reaction mixture was stirred at room temperature for 3 h. Then, the reaction was quenched with sat. NH_4Cl (10 mL) and diluted with EtOAc (20 mL), deionized water (20 mL). The organic layer was washed with saturated brine (10 mL), dried over Na_2SO_4 and concentrated *in vacuo*. The crude residue was purified by flash column chromatography (3:2 hexane:EtOAc) to afford **16** as a colorless oil (580 mg, 1.08 mmol) in 69% yield over 2 steps. ^{1}H NMR (400 MHz, $CDCl_3$) δ 7.70 (td, *J* = 7.9, 1.6 Hz, 4H), 7.53 – 7.36 (m, 6H), 6.97 – 6.76 (m, 6H), 5.77 (s, 1H), 5.61 (d, *J* = 6.0 Hz, 1H), 4.07 – 3.92 (m, 2H), 3.83 (s, 3H), 3.77 (s, 3H), 3.65 (q, *J* = 6.3 Hz, 1H), 1.13 (s, 9H). ^{13}C NMR (101 MHz, $CDCl_3$) δ 154.3, 154.0, 146.7, 145.5, 135.7, 135.6, 133.4, 133.2, 129.92, 129.89, 128.2, 127.9, 118.9, 114.4, 114.1, 111.1, 109.5, 108.4, 87.2, 66.1, 56.1, 55.9, 54.1, 26.9, 19.4. HRMS: m/z (ESI) Calcd for $C_{33}H_{36}NaO_5Si$ $[M+Na]^+$: 563.2224, found: 563.2253.

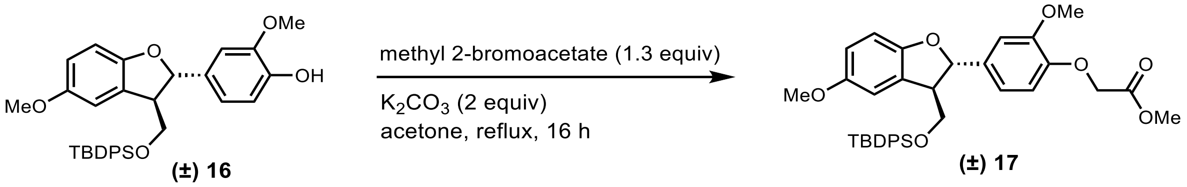

Methyl 2-(4-(3-(((tert-butyldiphenylsilyl)oxy)methyl)-5-methoxy-2,3-dihydrobenzofuran-2-yl)-2-methoxyphenoxy)acetate (17). A flame-dried 250 mL round bottom flask equipped with a stir bar was charged with **16** (362 mg, 0.67 mmol, 1 equiv), methyl 2-bromoacetate (0.08 mL, 0.872 mmol, 1.3 equiv), K_2CO_3 (185 mg, 1.34 mmol, 2 equiv), and acetone (10 mL). After purging with N_2, the reaction was heated to reflux in an oil bath and stirred at reflux for 16 h. The resulting reaction mixture was cooled to room temperature and filtered through a pad of Celite. The filtrate was evaporated to afford **17** as a colorless liquid (0.391g, 0.64 mmol) in 85% yield. ^{1}H NMR (400 MHz, $CDCl_3$) δ 7.626 (tt, *J* = 7.9, 1.5 Hz, 4H), 7.467 – 7.327 (m, 6H), 6.928 – 6.690 (m, 6H), 5.567 (d, *J* = 5.6 Hz, 1H), 4.682 (s, 2H), 3.941 (dd, *J* = 10.2, 5.6 Hz, 1H), 3.866 (dd, *J* = 10.2, 7.6 Hz, 1H), 3.794 (s, 3H), 3.787 (s, 3H), 3.715 (s, 3H), 1.055 (s, 9H).^{13}C NMR (101 MHz, $CDCl_3$) δ 169.6, 154.4, 154.0,

149.9, 146.9, 136.7, 135.73, 135.69, 133.5, 133.2, 129.99, 129.96, 128.0, 127.9, 118.0, 114.3, 114.2, 111.1, 109.6, 109.5, 86.9, 66.7, 66.2, 56.1, 56.0, 54.2, 52.4, 27.0, 19.4. HRMS: m/z (ESI) Calcd for $C_{36}H_{40}KO_7Si$ $[M+K]^+$: 651.2175, found: 651.2133.

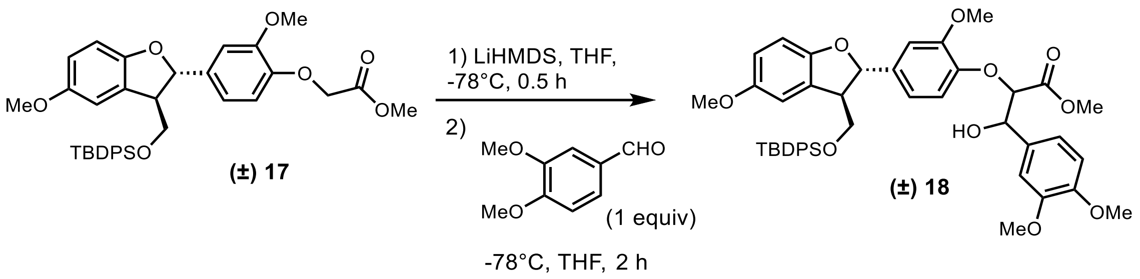

Methyl 2-(4-(3-(((tert-butyldiphenylsilyl)oxy)methyl)-5-methoxy-2,3-dihydrobenzofuran-2-yl)-2-methoxyphenoxy)-3-(3,4-dimethoxyphenyl)-3-hydroxypropanoate (18). In a N_2-filled glove box, LiHMDS (141 mg, 0.841 mmol, 1.2 equiv) was added to a flame-dried 250 mL three-neck round bottom flask equipped with a stir bar. The flask was then taken out of the glove box and THF (10 mL) was added *via* syringe. The reaction was cooled to -78 °C in an acetone/dry ice bath and a solution of **17** (430 mg, 0.701 mmol, 1.0 equiv) in THF (10 mL) was added dropwise over 15 min using a syringe pump. The reaction mixture was stirred for 1 h at -78 °C, after which a solution of 3,4-dimethoxybenzaldehyde (233 mg, 1.40 mmol, 2 equiv) in 5 mL THF was added dropwise. After 4 hrs at -78 °C, the reaction was quenched with a solution of saturated aqueous NH_4Cl and was allowed to warm to room temperature. The organic layer was separated and the aqueous phase was extracted with EtOAc (3 x 20 mL). The combined organic layers were dried over anhydrous Na_2SO_4, filtered and concentrated *in vacuo*. The crude residue was purified by flash column chromatography (3:1 hexanes:EtOAc) to afford **18** as a yellow liquid (0.425 g, 0.54 mmol) in 77% yield. Ratio of diastereomers (minor to major): 1:1.8, determined by ^1H NMR spectroscopy. Spectroscopic data are reported for the mixture of isomers. ^1H NMR (400 MHz, $CDCl_3$) δ 7.62 (tt, *J* = 8.1, 1.4 Hz, 4H), 7.45 – 7.32 (m, 6H), 7.04 – 6.67 (m, 9H), 5.56 (d, *J* = 5.6 and d, *J* = 2.9 Hz, 1H), 5.13 and 5.07 (d, *J* = 5.1 Hz and d, *J* = 6.9 Hz, 1H), 4.71 and 4.47 (d, *J* = 5.1 Hz and dd, *J* = 7.0, 1.9 Hz, 1H), 3.93 (dtd, *J* = 10.3, 4.9, 1.7 Hz, 2H), 3.89 – 3.58 (m, 12 H), 3.53 (h, *J* = 4.9 Hz, 1H), 1.05 (s, 9H). ^{13}C NMR (101 MHz, $CDCl_3$) δ 170.0, 169.9, 154.3, 153.9, 150.73, 150.68, 150.5, 150.4, 149.1, 149.0, 148.9, 148.8, 146.8, 146.7, 138.2, 138.2, 138.1, 135.71, 135.66, 133.3, 133.1, 131.6, 130.5, 130.00, 129.98, 127.92, 127.87, 127.80, 127.78, 119.5, 119.2, 118.8, 118.7, 118.3, 118.2, 118.0, 114.1, 111.1, 110.7, 109.9, 109.8, 109.6, 109.5, 86.7, 86.0, 85.5, 84.1, 84.0, 74.9, 73.9, 66.1, 56.1, 56.0, 55.95,

55.92, 54.22, 54.20, 52.4, 52.3, 26.9, 19.4. HRMS: m/z (ESI) Calcd for $C_{45}H_{50}KO_{10}Si$ $[M+K]^+$: 817.2805, found: 817.2885.

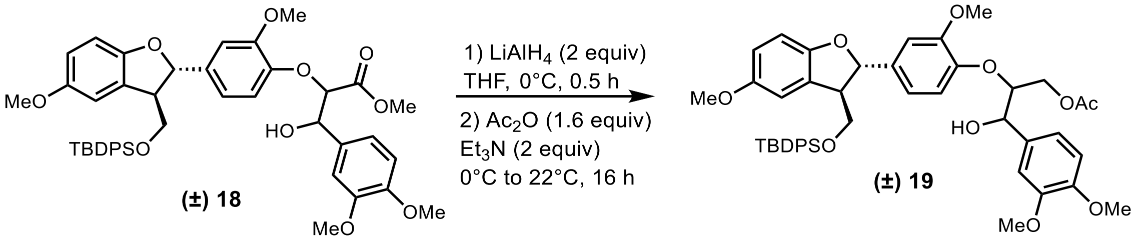

2-(4-(3-(((tert-butyldiphenylsilyl)oxy)methyl)-5-methoxy-2,3-dihydrobenzofuran-2-yl)-2-methoxyphenoxy)-3-(3,4-dimethoxyphenyl)-3-hydroxypropyl acetate (19). In a flame dried 100 mL flask equipped with a stir bar, $LiAlH_4$ (49 mg, 1.3 mmol, 2 equiv) was combined with diethyl ether (10 mL). The reaction mixture was cooled to 0 °C in an ice bath and a solution of **18** (499 mg, 0.64 mmol, 1 equiv) in THF (5 mL) was added dropwise. Then, the reaction mixture was stirred at room temperature for 30 min. The reaction was diluted with ethyl acetate (30 mL), followed by Fieser method workup. The organic layer was washed with saturated brine (30 mL), dried over anhydrous Na_2SO_4 and concentrated *in vacuo*. The crude residue was purified by flash column chromatography (3:7 hexanes:EtOAc) to afford 2-(4-(3-(((tert-butyldiphenylsilyl)oxy)methyl)-5-methoxy-2,3-dihydrobenzofuran-2-yl)-2-methoxyphenoxy)-1-(3,4-dimethoxyphenyl)propane-1,3-diol as a colorless oil (320 mg, 0.43 mmol).

In a flame dried 50 mL flask equipped with a stir bar, 2-(4-(3-(((tert-butyldiphenylsilyl)oxy)methyl)-5-methoxy-2,3-dihydrobenzofuran-2-yl)-2-methoxyphenoxy)-1-(3,4-dimethoxyphenyl)propane-1,3-diol (320 mg, 0.43 mmol, 1.0 equiv) was dissolved in a 1:1 mixture of THF: DCM (8 mL), triethyl amine (0.12 mL, 0.86 mmol, 2 equiv) was added, and the reaction was kept at 0 °C in an ice bath and then acetic anhydride (65 µL, 0.68 mmol, 1.6 equiv) was added slowly and then the reaction mixture was stirred overnight at room temperature. The resulting mixture was diluted with 20 mL deionized H_2O and 30 mL DCM, followed by phase separation. The aqueous phase was extracted with DCM (3 x 20 mL) and the organic layers were combined, dried over Na_2SO_4, filtered, and concentrated *in vacuo*. The crude residue was purified by flash column chromatography (2:3 hexane:EtOAc) to afford **19** as a colorless liquid (260 mg, 0.33 mmol) in 51% yield over two steps. Ratio of diastereomers (minor to major): 1:1.4, determined by ^1H NMR spectroscopy. Spectroscopic data are reported for the mixture of isomers. ^1H NMR (400 MHz, $CDCl_3$) δ 7.69 – 7.58 (m, 4H), 7.49 – 7.32 (m, 6H), 7.11 – 6.67 (m, 9H), 5.60 (d, *J* = 5.5 Hz, 1H), 4.88 and

4.85 (d, *J* = 3.3 Hz and d, *J* = 8.0 Hz, 1H), 4.43 – 4.33 and 4.24 – 4.08 (m and m, 2H), 4.02 and 3.96 (dd, *J* = 11.8, 5.4 Hz and ddd, *J* = 10.1, 5.6, 1.0 Hz, 2H), 3.92 – 3.69 (m, 12H), 3.64 – 3.50 (m, 1H), 2.05 and 2.03 (s and s, 3H), 1.06 (s, 9H). ^{13}C NMR (101 MHz, $CDCl_3$) δ 171.1, 170.7, 154.4, 154.0, 151.8, 151.7, 151.1, 151.0, 149.3, 149.2, 149.1, 148.6, 147.8, 146.64, 146.60, 138.41, 138.39, 138.34, 138.29, 135.73, 135.68, 133.4, 133.2, 131.8, 131.6, 131.5, 130.02, 130.00, 127.9, 127.83, 127.81, 120.9, 120.8, 120.5, 120.4, 119.9, 118.8 118.7, 118.63, 118.58, 114.2, 111.2, 111.1, 109.9, 109.8, 109.7, 109.59, 109.56, 109.4, 86.89, 86.87, 86.82, 86.79, 86.6, 86.5, 84.7, 74.4, 72.1, 72.0, 66.24, 66.21, 63.4, 62.9, 56.13, 56.10, 56.04, 56.03, 55.97, 54.31, 54.28, 54.2, 27.0, 21.0, 20.9, 19.4. HRMS: m/z (ESI) Calcd for $C_{46}H_{52}NaO_{10}Si$ $[M+Na]^+$: 815.3222, found: 815.3245.

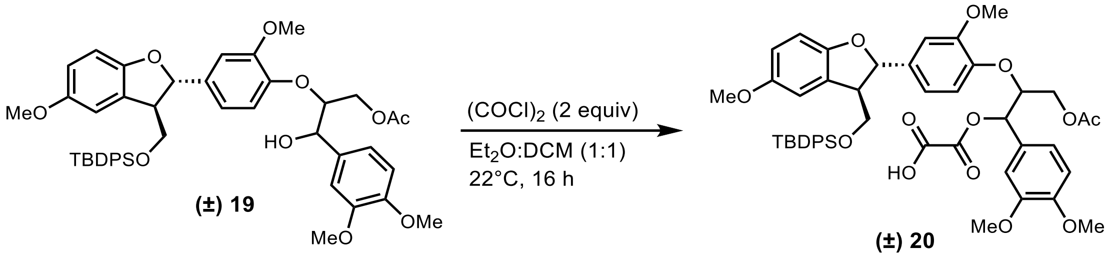

2-(3-acetoxy-2-(4-(3-(((tert-butyldiphenylsilyl)oxy)methyl)-5-methoxy-2,3-dihydrobenzofuran-2-yl)-2-methoxyphenoxy)-1-(3,4-dimethoxyphenyl)propoxy)-2-oxoacetic acid (20). In a flame dried 50 mL round bottom flask equipped with a stir bar, **19** (260 mg, 0.33 mmol, 1.0 equiv) was dissolved in a 1:1 mixture of Et_2O:DCM (5 mL). Oxalyl chloride (58 µL, 0.66 mmol, 2 equiv) was added slowly, and the reaction mixture stirred at room temperature for 16 h. Then, the reaction was carefully treated with water (10 mL). The biphasic reaction mixture was stirred vigorously for 1 hour and the layers were separated. The aqueous phase was extracted with Et_2O (3 x 10 mL) and the combined ethereal extracts were washed with water (2 x 10 mL) and brine (10 mL), dried over Na_2SO_4 filtered, and concentrated *in vacuo*. The crude residue was purified by flash column chromatography (95:5 DCM:MeOH) to afford **20** as a colorless liquid (102 mg, 0.11 mmol) in 37% yield. Ratio of diastereomers (minor to major): 1:1.4, determined by ^{1}H NMR spectroscopy. Spectroscopic data are reported for the mixture of isomers. ^{1}H NMR (400 MHz, DMSO) δ 7.56 (ddt, *J* = 15.5, 6.6, 1.6 Hz, 4H), 7.41 (tdd, *J* = 14.6, 8.1, 4.1 Hz, 6H), 7.03 – 6.71 (m, 8H), 5.93 and 5.89 (d, *J* = 5.4 Hz and d, *J* = 5.4 Hz, 1H), 5.48 (d, *J* = 6.1 Hz, 1H), 4.82 – 4.71 (m, 1H), 4.26 – 3.99 (m, 2H), 3.99 – 3.83 (m, 2H), 3.74 – 3.63 (m, 3H), 3.36 (s, 9H), 1.92 and 1.89 (s and s, 3H), 0.96 (s, 9H). ^{13}C NMR (101 MHz, DMSO) δ 170.2, 170.1, 170.0, 161.7, 153.8, 153.4, 150.13, 150.11, 150.0, 148.6, 148.5, 148.4, 147.1, 146.4, 135.9, 135.7, 135.6, 135.10, 135.07, 132.8, 132.6, 130.0, 129.9,

129.2, 129.1, 128.2, 128.1, 127.93, 127.89, 119.6, 119.5, 117.80, 117.76, 117.4, 116.8, 113.9, 111.4, 111.2, 110.92, 110.87, 110.3, 110.2, 109.0, 85.9, 78.9, 78.7, 72.2, 67.0, 65.3, 64.9, 62.6, 62.3, 55.6, 55.55, 55.47, 55.43, 55.41, 55.3, 54.9, 53.0, 40.1, 39.9, 39.7, 39.5, 39.3, 39.1, 39.0, 26.6, 26.5, 26.3, 25.1, 20.4, 18.8, 15.2, 14.1. HRMS: m/z (ESI) Calcd for $C_{48}H_{52}KO_{13}Si$ $[M+K]^+$: 903.2809, found: 903.2777.

6. NMR Spectra

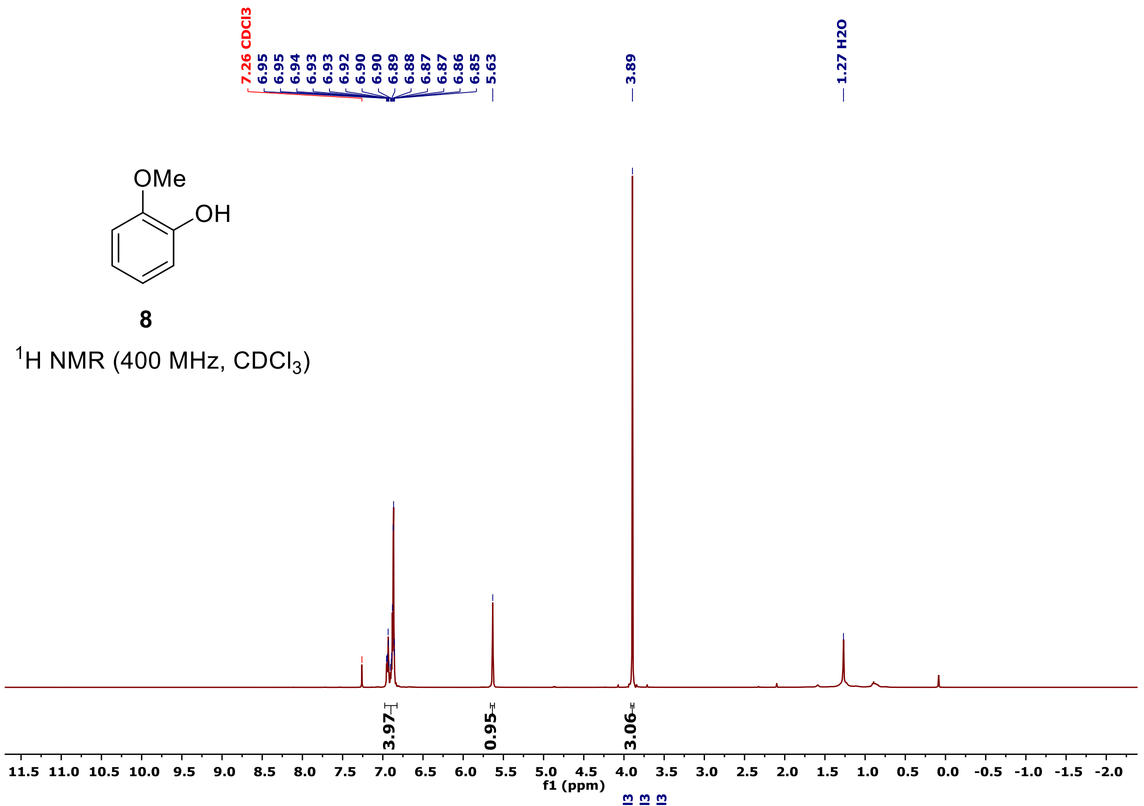

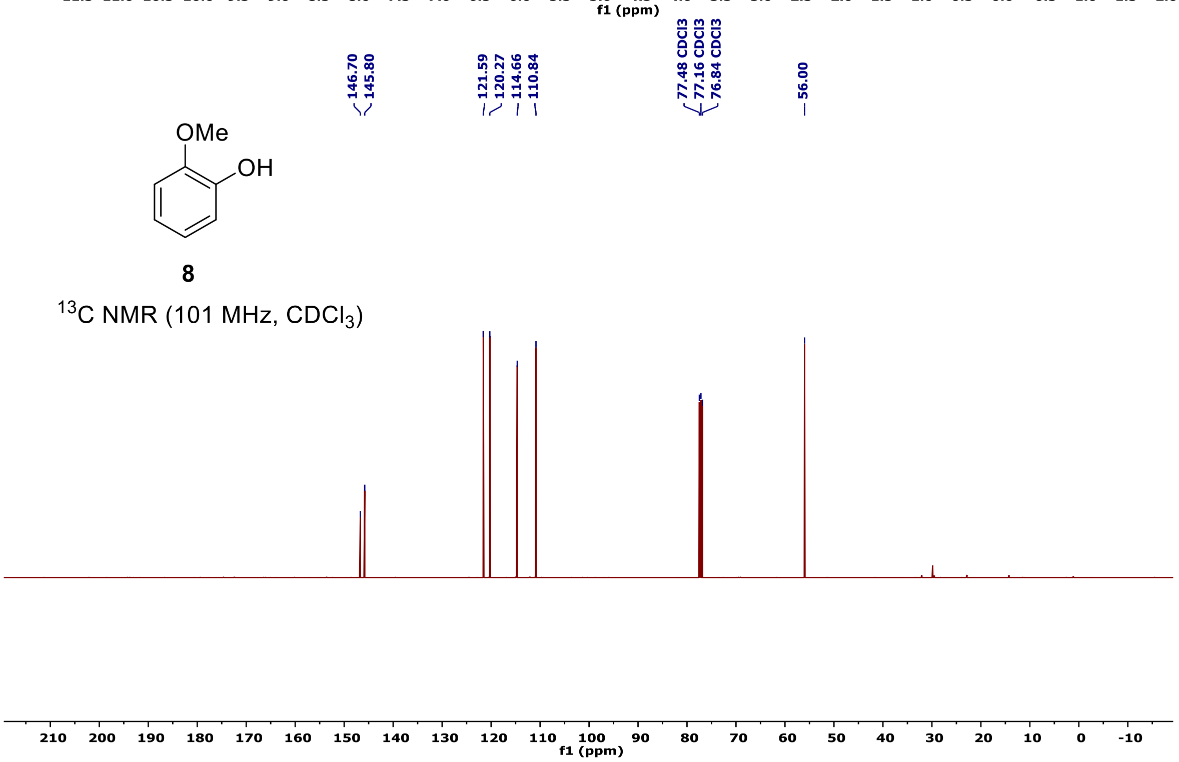

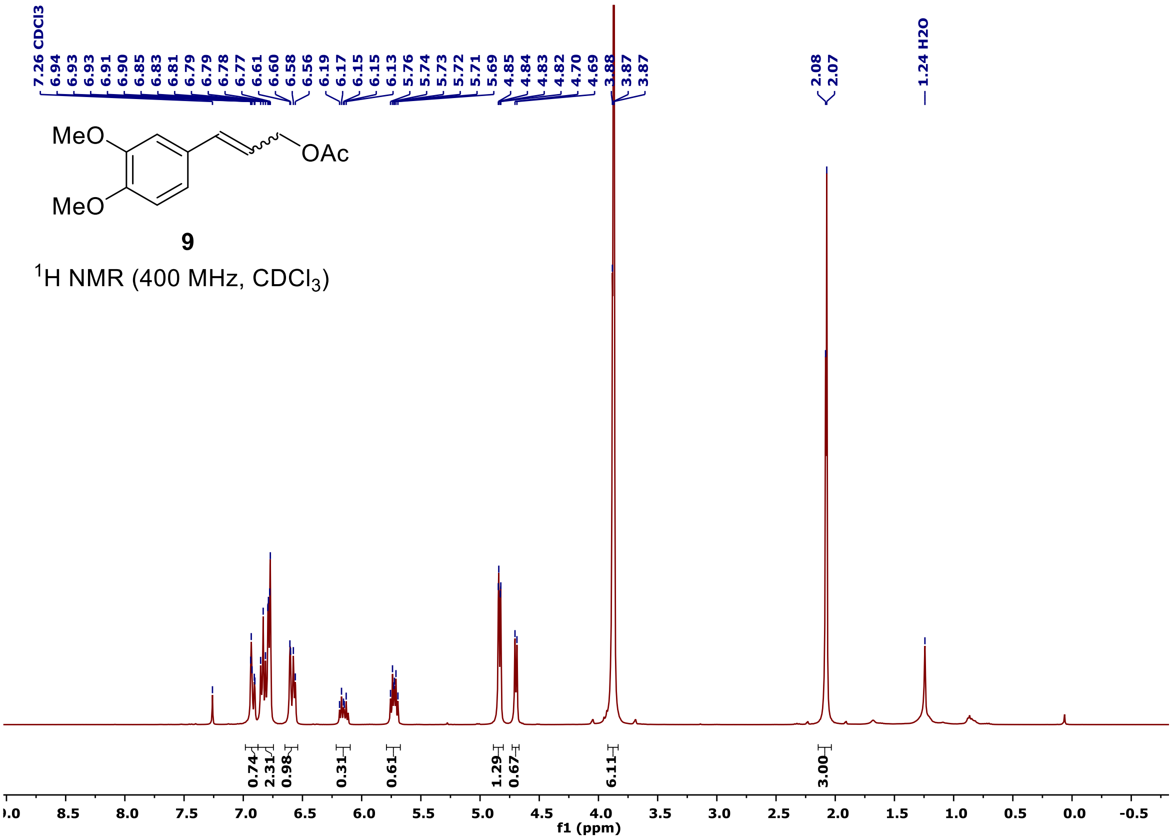
7.26 CDCl3
6.94
6.93
6.93
6.91
6.90
6.85
6.83
6.81
6.79
6.79
6.78
6.77
6.61
6.60
6.58
6.56
6.19
6.17
6.15
6.15
6.13
5.76
5.74
5.73
5.72
5.71
5.69
4.85
4.84
4.83
4.82
4.70
4.69
3.88
3.87
3.87
2.08
2.07
1.24 H2O
MeO
MeO
OAc
9
1H NMR (400 MHz, CDCl3)
0.74
2.31
0.98
0.31
0.61
1.29
0.67
6.11
3.00
8.5
8.0
7.5
7.0
6.5
6.0
5.5
5.0
4.5
4.0
3.5
3.0
2.5
2.0
1.5
1.0
0.5
0.0
-0.5
f1 (ppm)

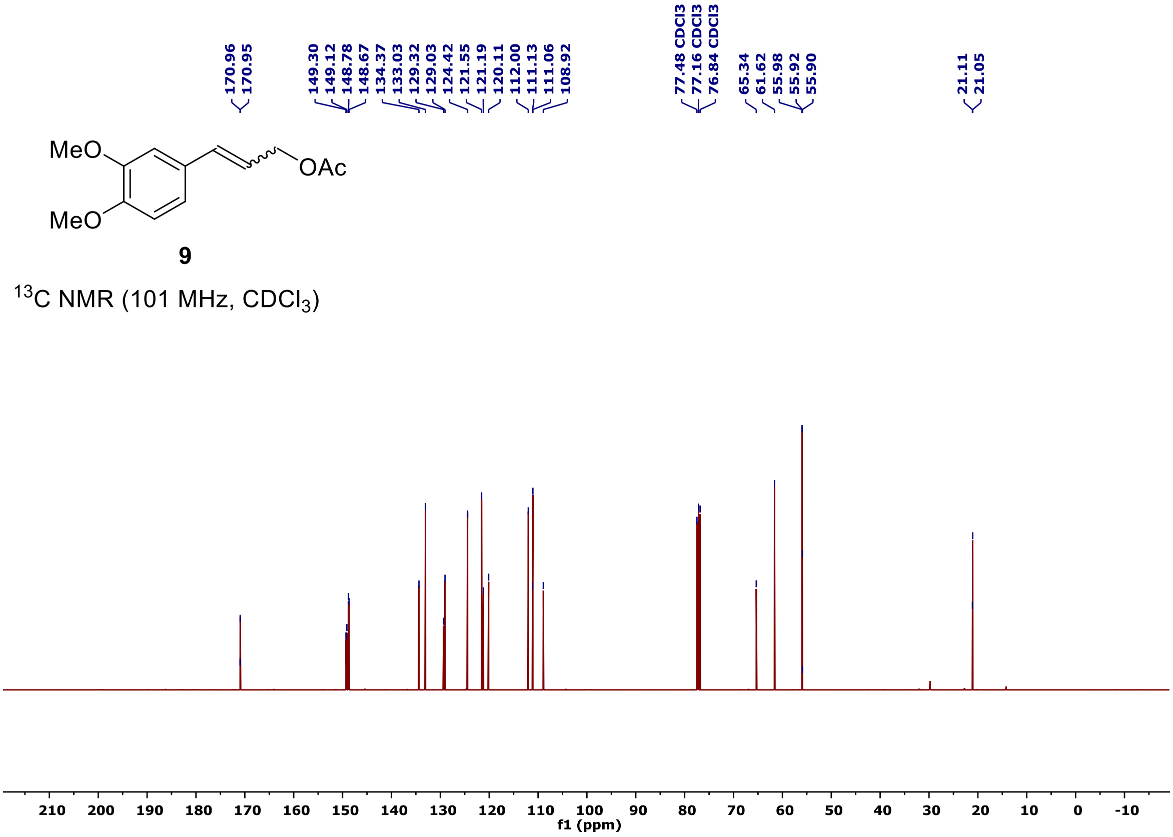
170.96
170.95
149.30
149.12
148.78
148.67
134.37
133.03
129.32
129.03
124.42
121.55
121.19
120.11
112.00
111.13
111.06
108.92
77.48 CDCl3
77.16 CDCl3
76.84 CDCl3
65.34
61.62
55.98
55.92
55.90
21.11
21.05
MeO
MeO
OAc
9
13C NMR (101 MHz, CDCl3)
210
200
190
180
170
160
150
140
130
120
110
100
90
80
70
60
50
40
30
20
10
0
-10
f1 (ppm)

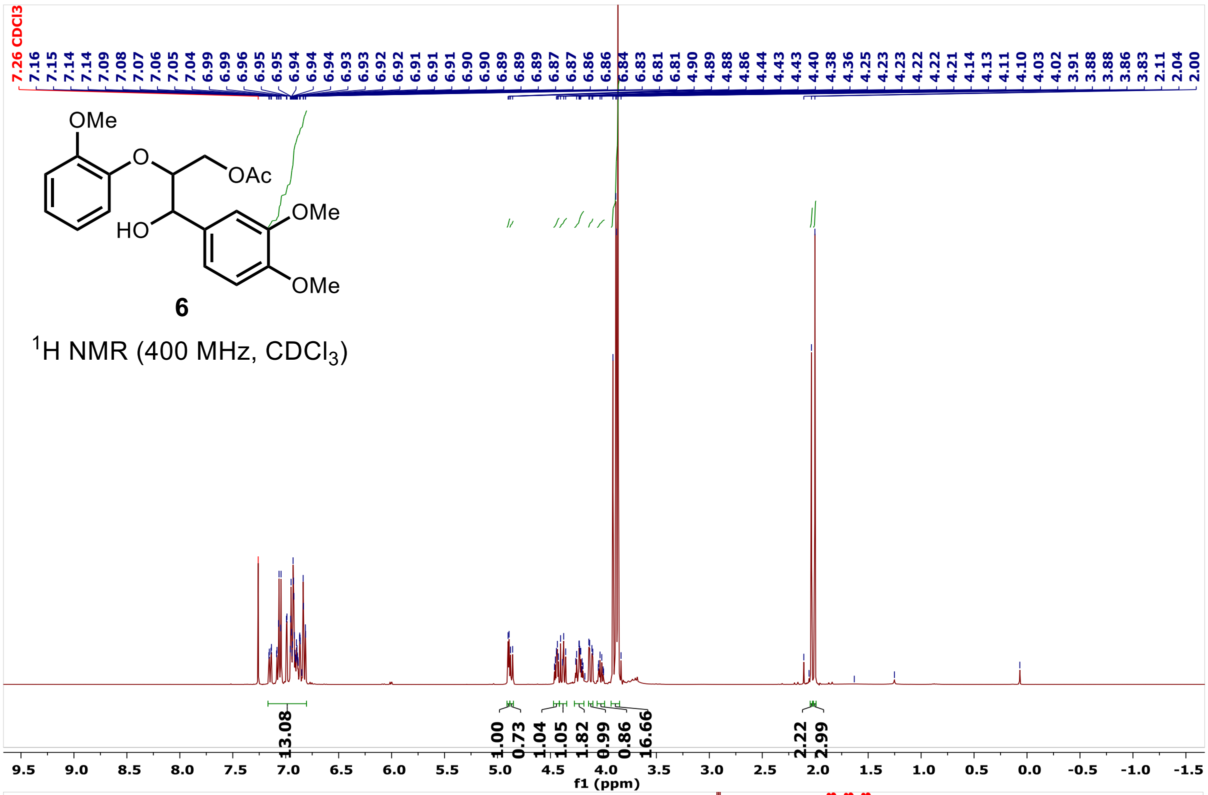
7.26 CDCl3
7.16 7.15 7.14 7.14 7.09 7.08 7.07 7.06 7.05 7.04 6.99 6.99 6.96 6.95 6.95 6.94 6.94 6.94 6.93 6.93 6.92 6.91 6.91 6.91 6.90 6.89 6.89 6.89 6.87 6.87 6.86 6.86 6.84 6.83 6.81 6.81 4.90 4.89 4.88 4.86 4.44 4.43 4.43 4.40 4.38 4.36 4.25 4.23 4.23 4.22 4.22 4.21 4.14 4.13 4.11 4.10 4.03 4.02 3.91 3.88 3.86 3.83 2.11 2.04 2.00
OMe
O
OAc
HO
OMe
OMe
6
^{1}H NMR (400 MHz, $CDCl_3$)
13.08
1.00
0.73
1.04
1.05
1.82
0.99
0.86
16.66
2.22
2.99
9.5 9.0 8.5 8.0 7.5 7.0 6.5 6.0 5.5 5.0 4.5 4.0 3.5 3.0 2.5 2.0 1.5 1.0 0.5 0.0 -0.5 -1.0 -1.5
f1 (ppm)

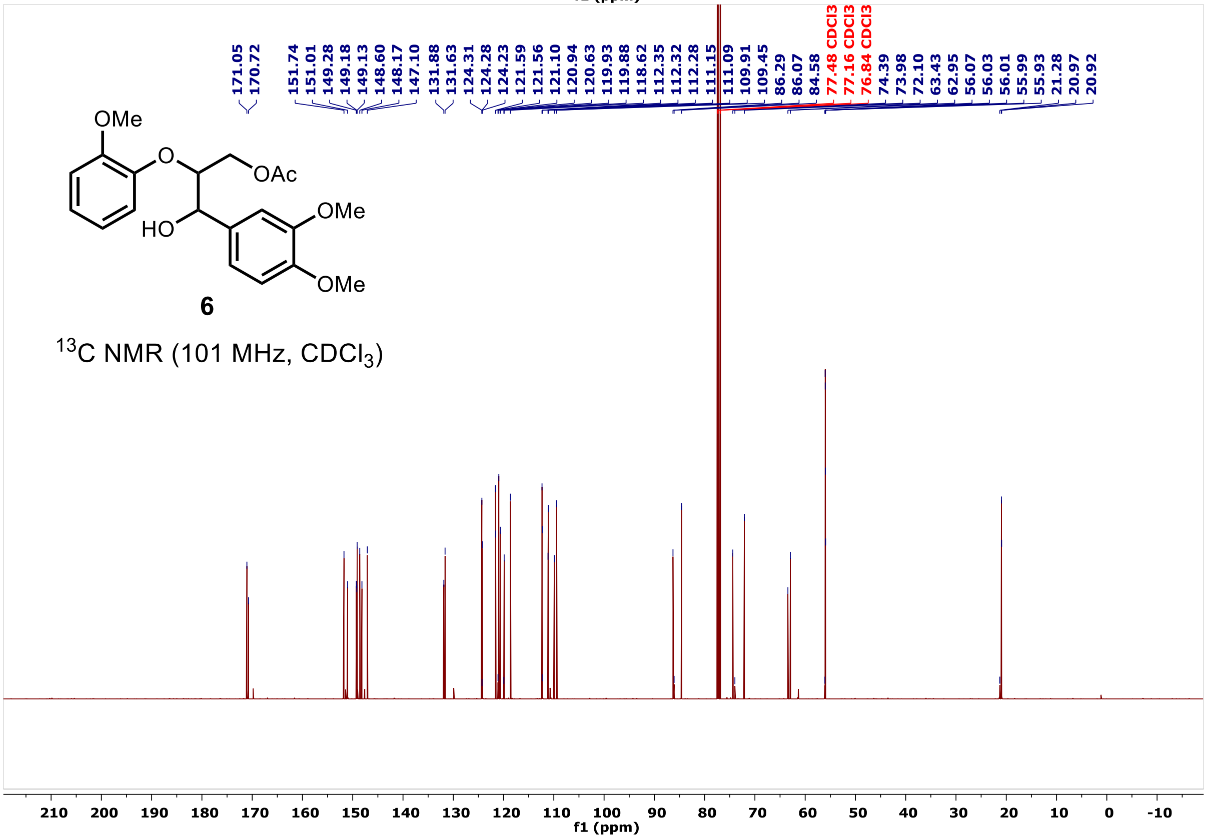
171.05 170.72 151.74 151.01 149.28 149.18 149.13 148.60 148.17 147.10 131.88 131.63 124.31 124.28 124.23 121.59 121.56 121.10 120.94 120.63 119.93 119.88 118.62 112.35 112.32 112.28 111.15 111.09 109.91 109.45 86.29 86.07 84.58
77.48 CDCl3 77.16 CDCl3 76.84 CDCl3
74.39 73.98 72.10 63.43 62.95 56.07 56.03 56.01 55.99 55.93 21.28 20.97 20.92
OMe
O
OAc
HO
OMe
OMe
6
^{13}C NMR (101 MHz, $CDCl_3$)
210 200 190 180 170 160 150 140 130 120 110 100 90 80 70 60 50 40 30 20 10 0 -10
f1 (ppm)

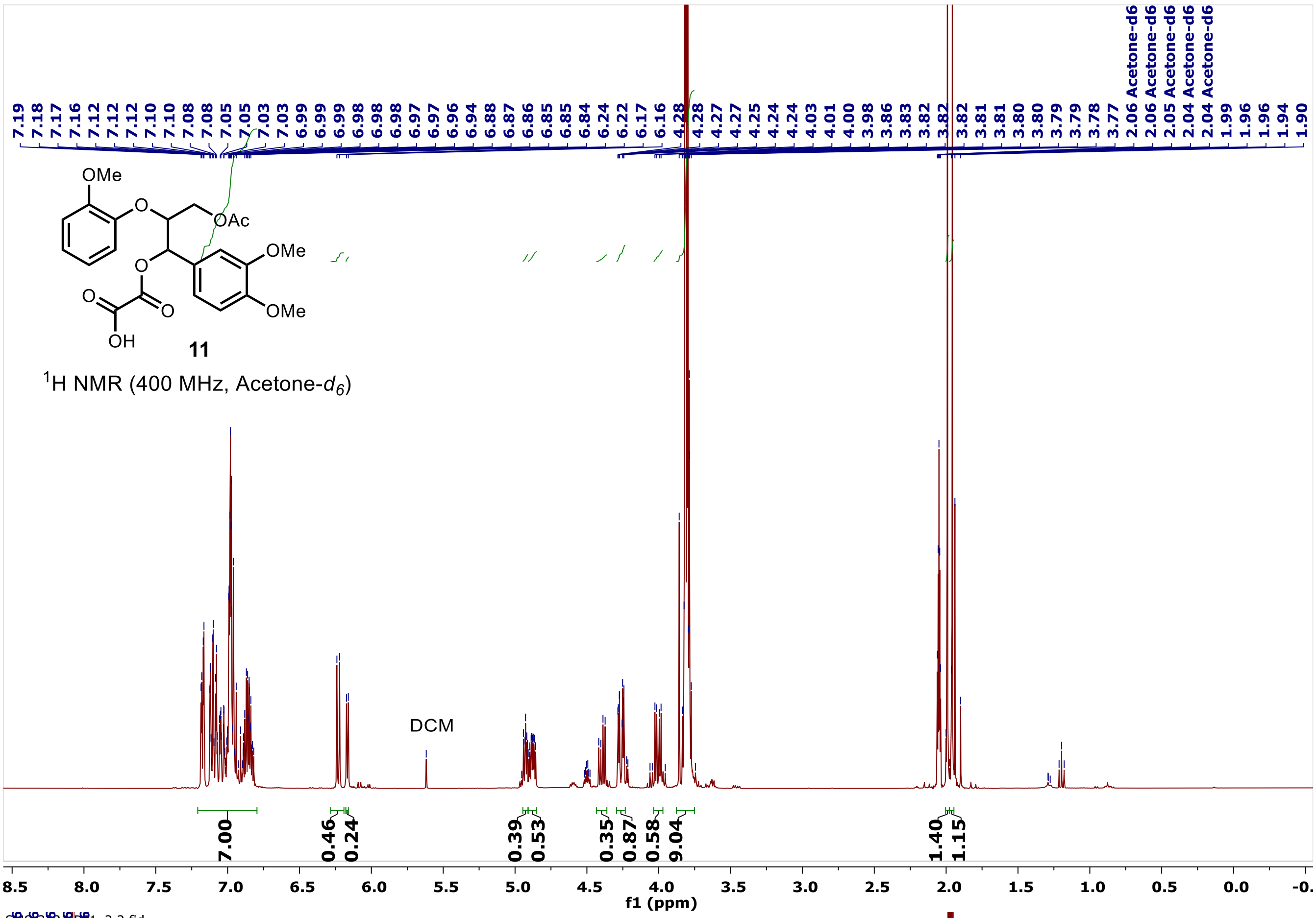
7.19 7.18 7.17 7.16 7.12 7.12 7.12 7.10 7.10 7.08 7.08 7.05 7.05 7.03 6.99 6.99 6.98 6.98 6.97 6.97 6.96 6.94 6.88 6.87 6.86 6.85 6.85 6.84 6.24 6.22 6.17 6.16 4.28 4.28 4.27 4.25 4.24 4.24 4.03 4.01 4.00 3.98 3.86 3.83 3.82 3.82 3.82 3.81 3.81 3.80 3.80 3.79 3.79 3.78 3.77
2.06 Acetone-d6 2.06 Acetone-d6 2.05 Acetone-d6 2.04 Acetone-d6 2.04 Acetone-d6
1.99 1.96 1.96 1.94 1.90
OMe
O
OAc
OMe
O
O
O
OH
OMe
11
^{1}H NMR (400 MHz, Acetone-d_6)
DCM
7.00 0.46 0.24 0.39 0.53 0.35 0.87 0.58 9.04 1.40 1.15
8.5 8.0 7.5 7.0 6.5 6.0 5.5 5.0 4.5 4.0 3.5 3.0 2.5 2.0 1.5 1.0 0.5 0.0 -0.
f1 (ppm)

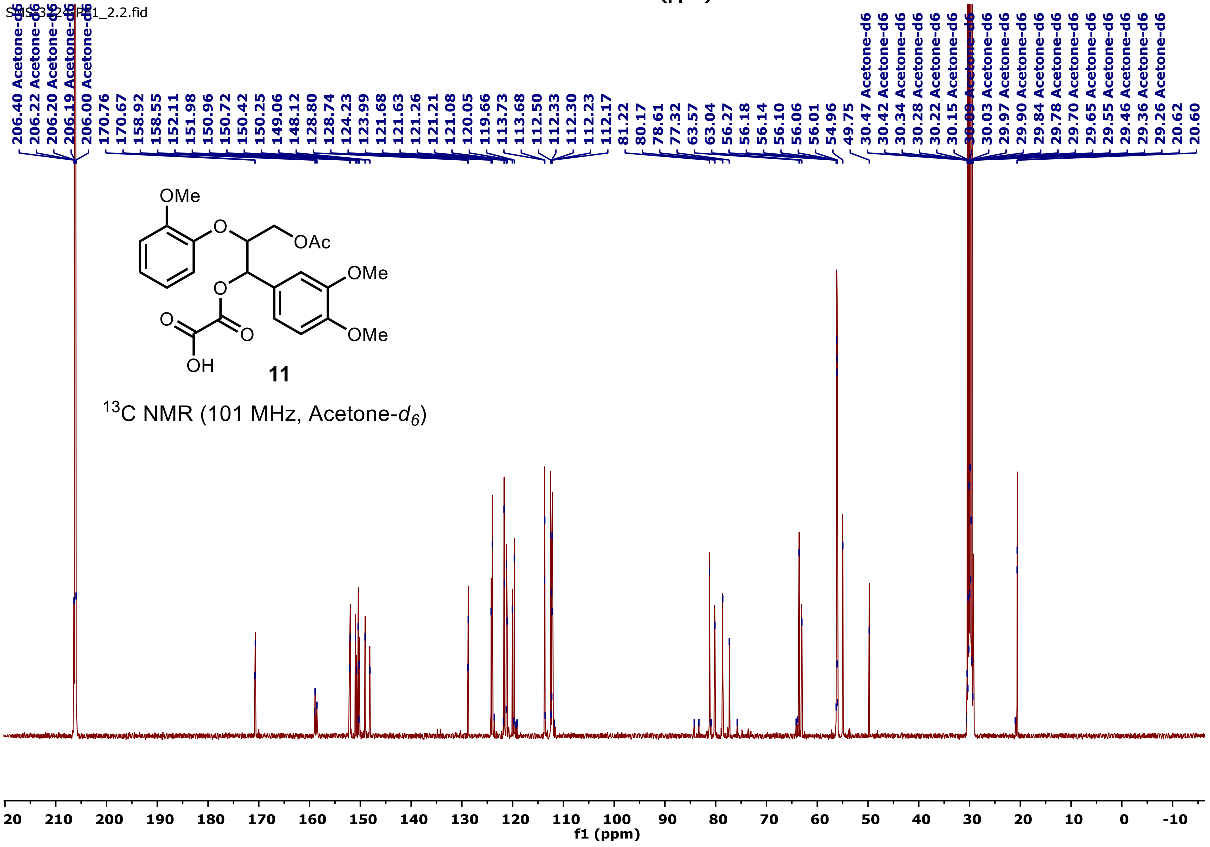
206.40 Acetone-d6 206.22 Acetone-d6 206.20 Acetone-d6 206.19 Acetone-d6 206.00 Acetone-d6
170.76 170.67 158.92 158.55 152.11 151.98 150.96 150.72 150.42 150.25 149.06 148.12 128.80 128.74 124.23 123.99 121.68 121.63 121.26 121.21 121.08 120.05 119.66 113.73 113.68 112.50 112.33 112.30 112.23 112.17 81.22 80.17 78.61 77.32 63.57 63.04 56.27 56.18 56.14 56.10 56.06 56.01 54.96 49.75
30.47 Acetone-d6 30.42 Acetone-d6 30.34 Acetone-d6 30.28 Acetone-d6 30.22 Acetone-d6 30.15 Acetone-d6 30.09 Acetone-d6 30.03 Acetone-d6 29.97 Acetone-d6 29.90 Acetone-d6 29.84 Acetone-d6 29.78 Acetone-d6 29.70 Acetone-d6 29.65 Acetone-d6 29.55 Acetone-d6 29.46 Acetone-d6 29.36 Acetone-d6 29.26 Acetone-d6
20.62 20.60
OMe
O
OAc
OMe
O
O
O
OH
OMe
11
^{13}C NMR (101 MHz, Acetone-d_6)
20 210 200 190 180 170 160 150 140 130 120 110 100 90 80 70 60 50 40 30 20 10 0 -10
f1 (ppm)

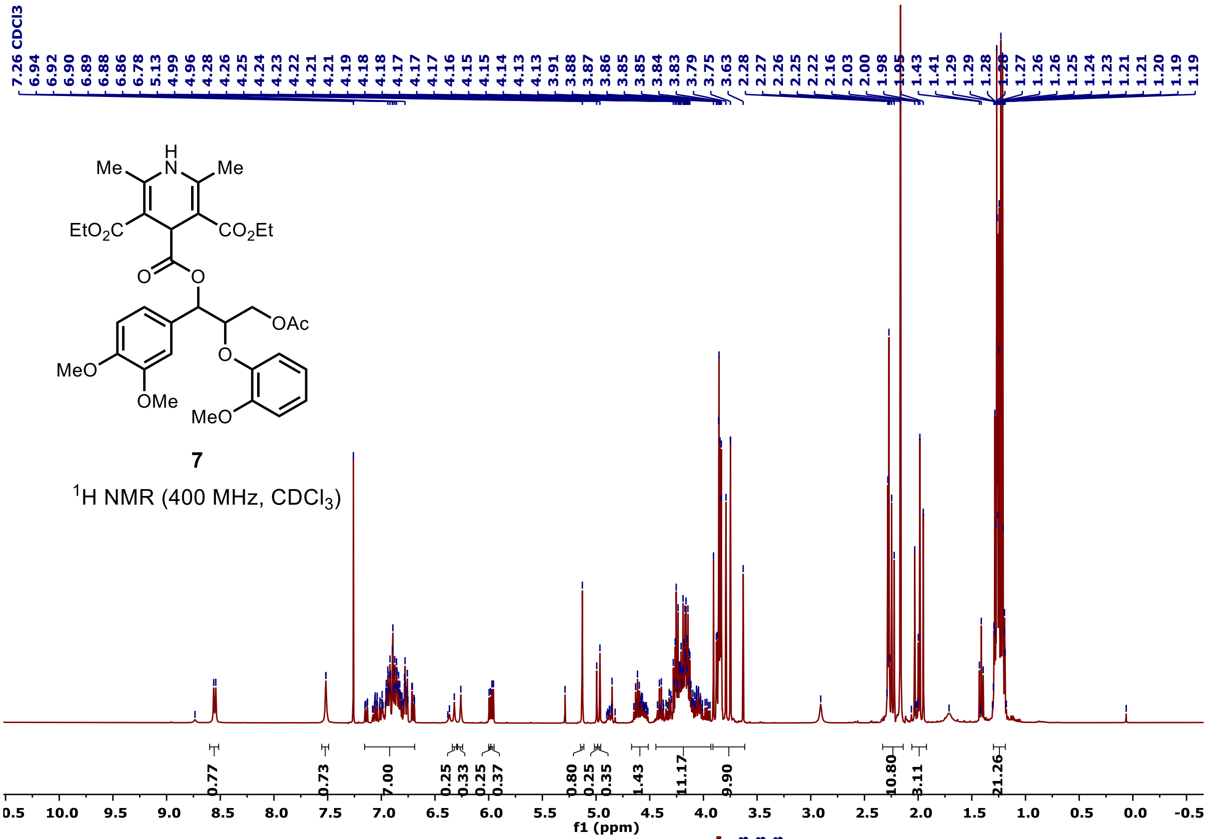

7

^{1}H NMR (400 MHz, $CDCl_3$)

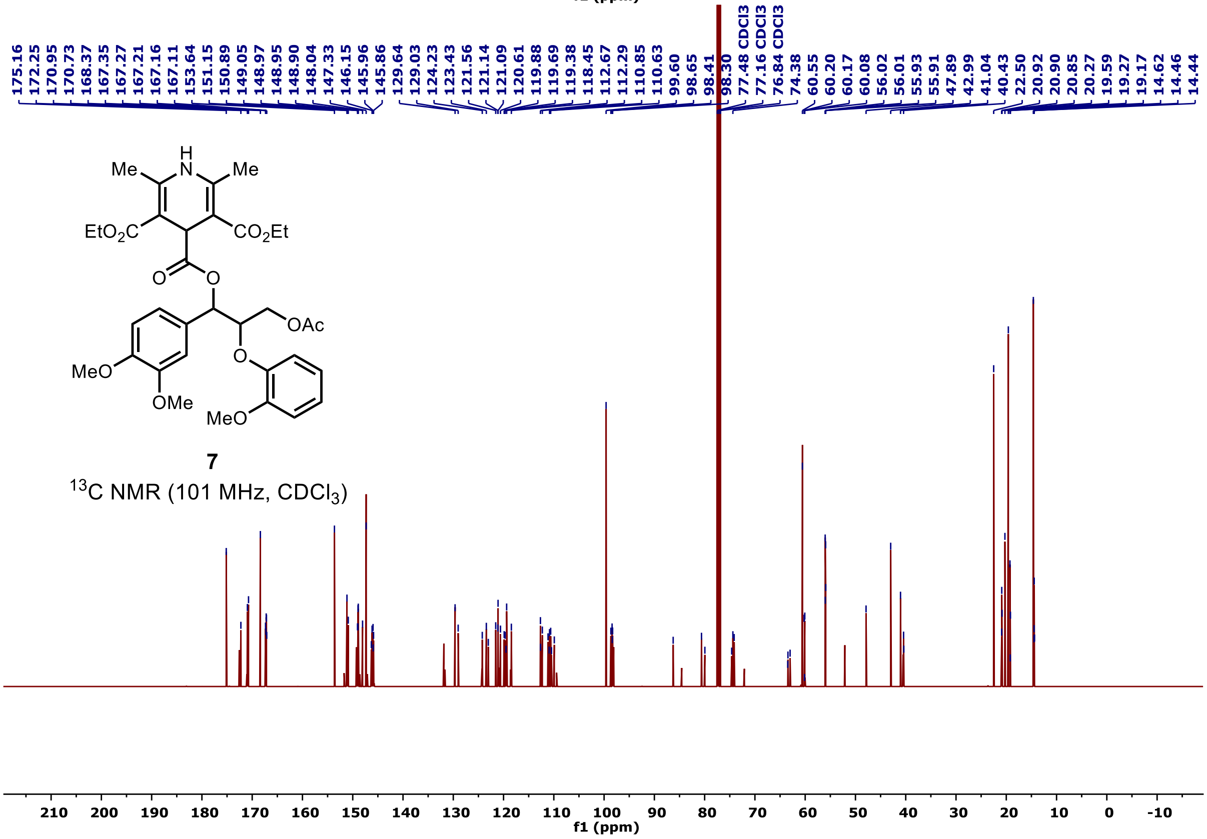

7

^{13}C NMR (101 MHz, $CDCl_3$)

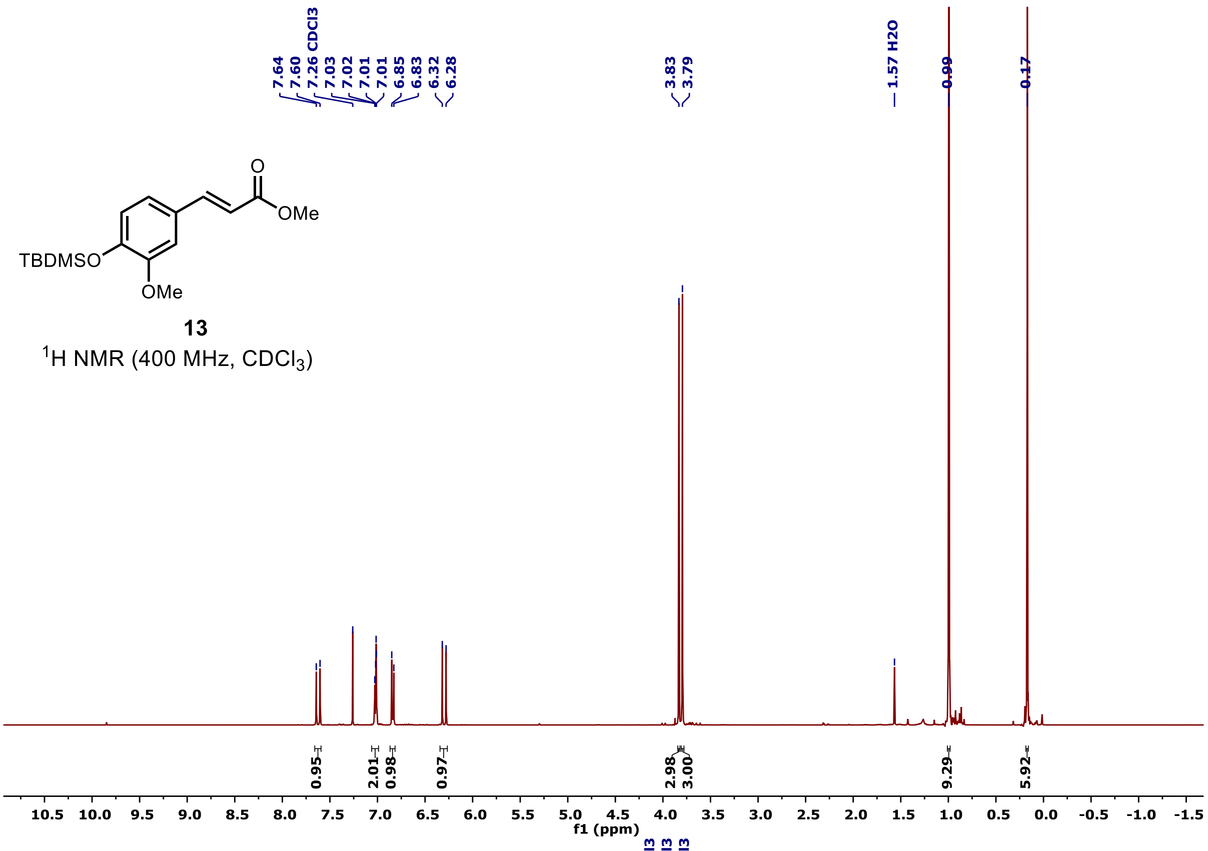
7.64
7.60
7.26 CDCl3
7.03
7.02
7.01
7.01
6.85
6.83
6.32
6.28
3.83
3.79
1.57 H2O
0.99
0.17
TBDMSO
OMe
OMe
O
13
^1H NMR (400 MHz, $CDCl_3$)
0.95
2.01
0.98
0.97
2.98
3.00
9.29
5.92
f1 (ppm)

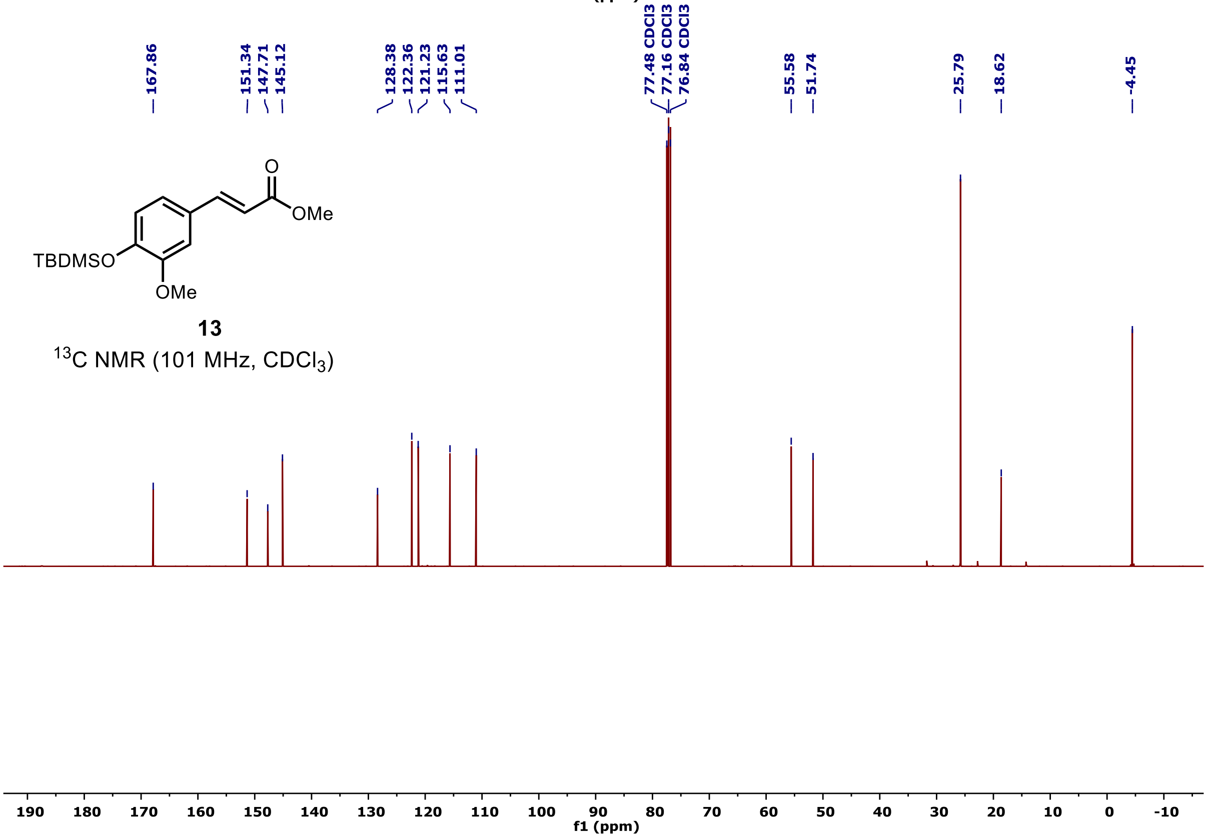
167.86
151.34
147.71
145.12
128.38
122.36
121.23
115.63
111.01
77.48 CDCl3
77.16 CDCl3
76.84 CDCl3
55.58
51.74
25.79
18.62
-4.45
TBDMSO
OMe
OMe
O
13
^{13}C NMR (101 MHz, $CDCl_3$)
f1 (ppm)

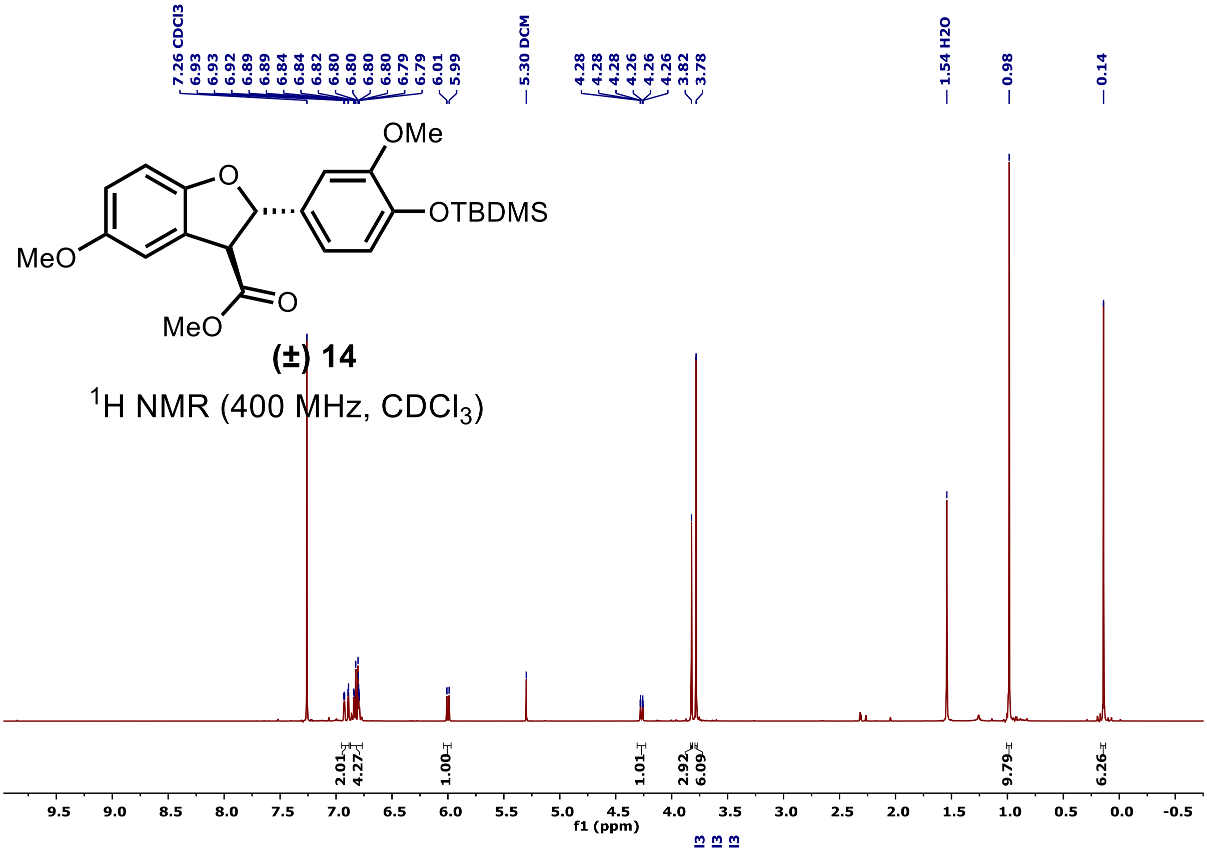
7.26 CDCl3
6.93
6.93
6.92
6.89
6.89
6.84
6.84
6.82
6.80
6.80
6.80
6.80
6.79
6.79
6.01
5.99
5.30 DCM
4.28
4.28
4.28
4.26
4.26
4.26
3.82
3.78
1.54 H2O
0.98
0.14
OMe
OTBDMS
MeO
O
MeO
O
(±) 14
1H NMR (400 MHz, CDCl3)
2.01
4.27
1.00
1.01
2.92
6.09
9.79
6.26
9.5
9.0
8.5
8.0
7.5
7.0
6.5
6.0
5.5
5.0
4.5
4.0
3.5
3.0
2.5
2.0
1.5
1.0
0.5
0.0
-0.5
f1 (ppm)

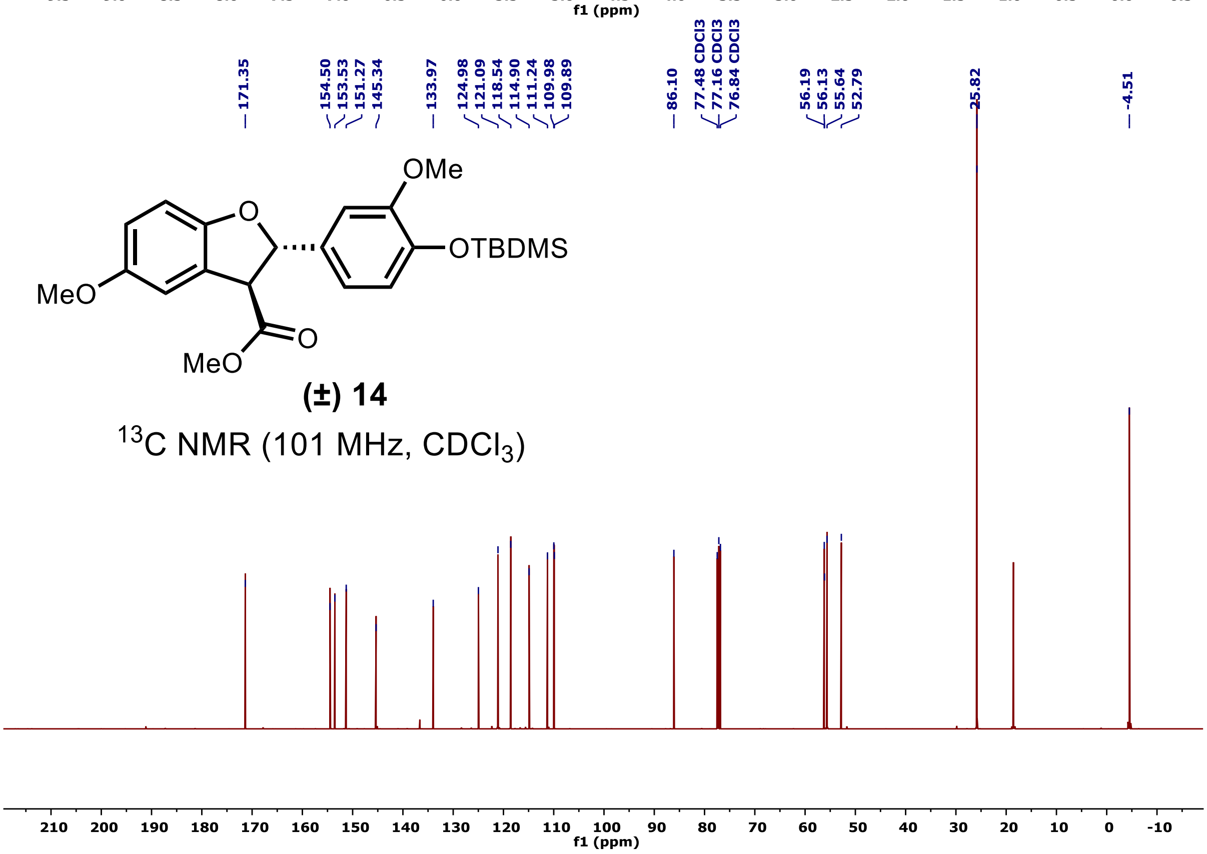
171.35
154.50
153.53
151.27
145.34
133.97
124.98
121.09
118.54
114.90
111.24
109.98
109.89
86.10
77.48 CDCl3
77.16 CDCl3
76.84 CDCl3
56.19
56.13
55.64
52.79
25.82
-4.51
OMe
OTBDMS
MeO
O
MeO
O
(±) 14
13C NMR (101 MHz, CDCl3)
210
200
190
180
170
160
150
140
130
120
110
100
90
80
70
60
50
40
30
20
10
0
-10
f1 (ppm)

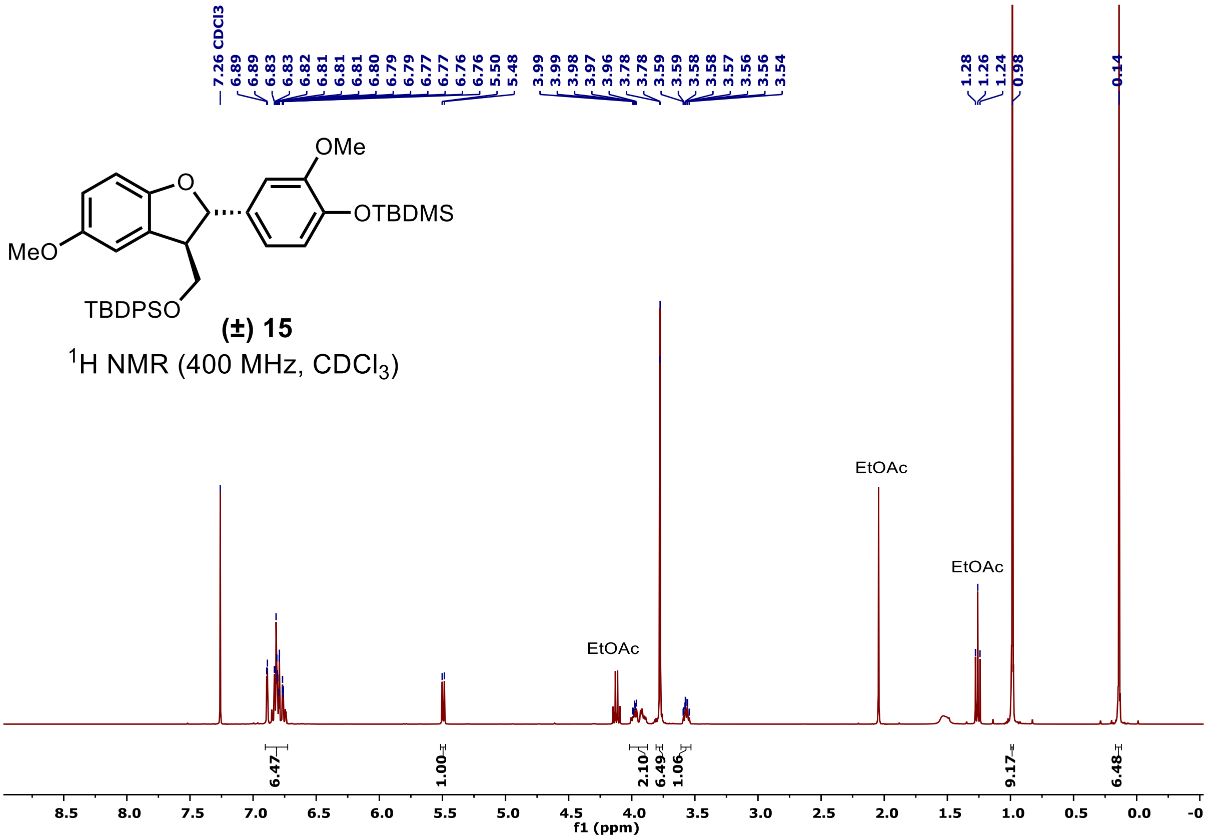
7.26 CDCl3
6.89
6.89
6.83
6.83
6.82
6.81
6.81
6.80
6.79
6.79
6.77
6.77
6.76
6.76
5.50
5.48
3.99
3.99
3.98
3.97
3.96
3.78
3.78
3.59
3.59
3.58
3.58
3.57
3.56
3.56
3.54
1.28
1.26
1.24
0.98
0.14
OMe
OTBDMS
MeO
TBDPSO
(±) 15
1H NMR (400 MHz, CDCl3)
EtOAc
EtOAc
EtOAc
6.47
1.00
2.10
6.49
1.06
9.17
6.48
f1 (ppm)

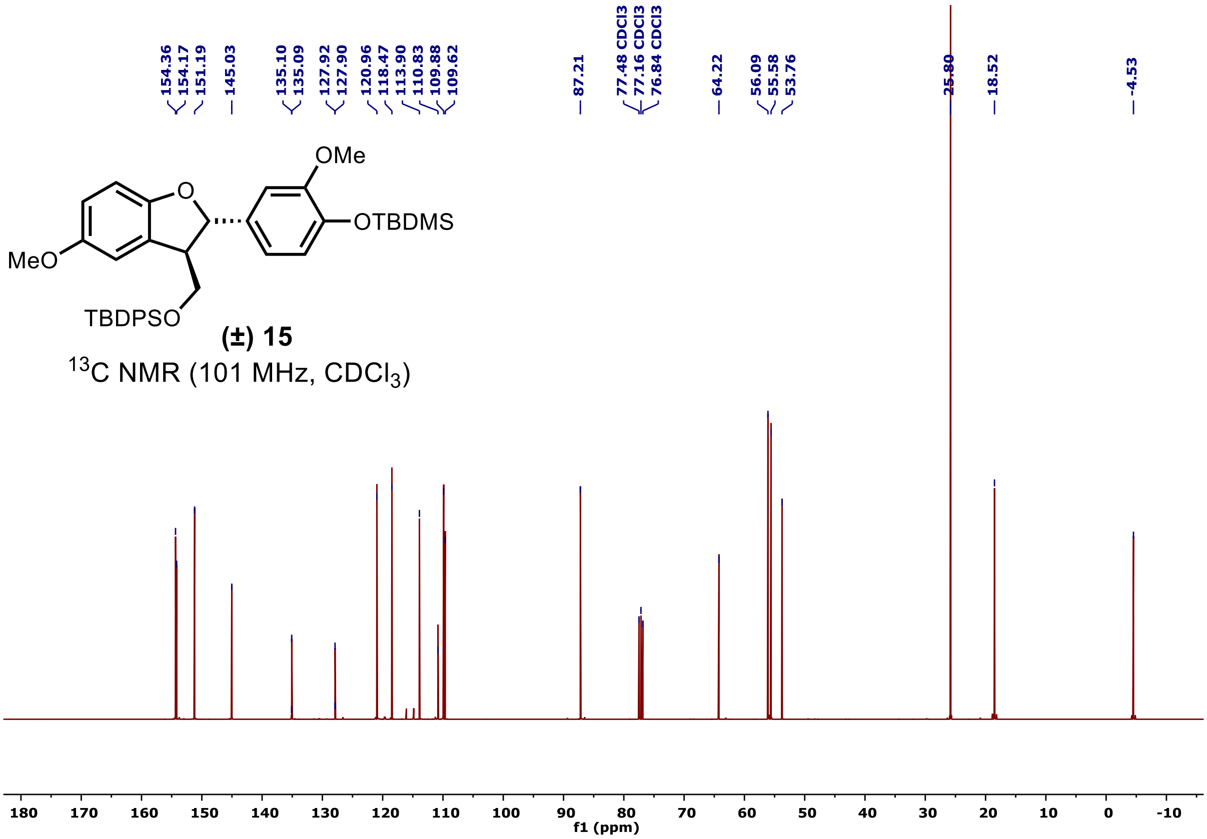
154.36
154.17
151.19
145.03
135.10
135.09
127.92
127.90
120.96
118.47
113.90
110.83
109.88
109.62
87.21
77.48 CDCl3
77.16 CDCl3
76.84 CDCl3
64.22
56.09
55.58
53.76
25.80
18.52
-4.53
OMe
OTBDMS
MeO
TBDPSO
(±) 15
13C NMR (101 MHz, CDCl3)
f1 (ppm)

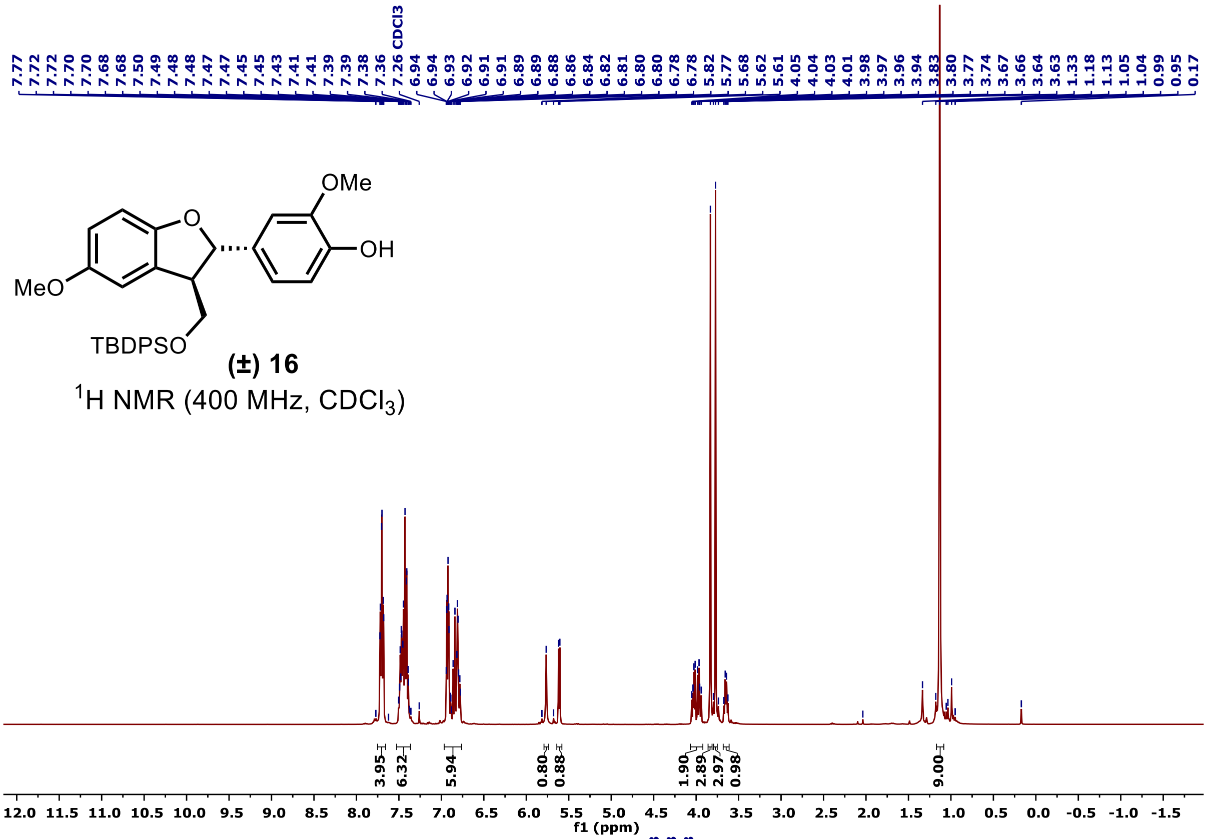
7.77 7.72 7.72 7.70 7.70 7.68 7.68 7.50 7.49 7.48 7.48 7.47 7.47 7.45 7.45 7.43 7.41 7.41 7.39 7.39 7.38 7.36 7.26 CDCl3 6.94 6.94 6.93 6.92 6.91 6.91 6.89 6.89 6.88 6.86 6.84 6.82 6.81 6.80 6.80 6.78 6.78 5.82 5.77 5.68 5.62 5.61 4.05 4.04 4.03 4.01 3.98 3.97 3.96 3.94 3.83 3.80 3.77 3.74 3.67 3.66 3.64 3.63 1.33 1.18 1.13 1.05 1.04 0.99 0.95 0.17
OMe
OH
MeO
TBDPSO
(±) 16
1H NMR (400 MHz, CDCl3)
3.95 6.32 5.94 0.80 0.88 1.90 2.89 2.97 0.98 9.00
12.0 11.5 11.0 10.5 10.0 9.5 9.0 8.5 8.0 7.5 7.0 6.5 6.0 5.5 5.0 4.5 4.0 3.5 3.0 2.5 2.0 1.5 1.0 0.5 0.0 -0.5 -1.0 -1.5
f1 (ppm)

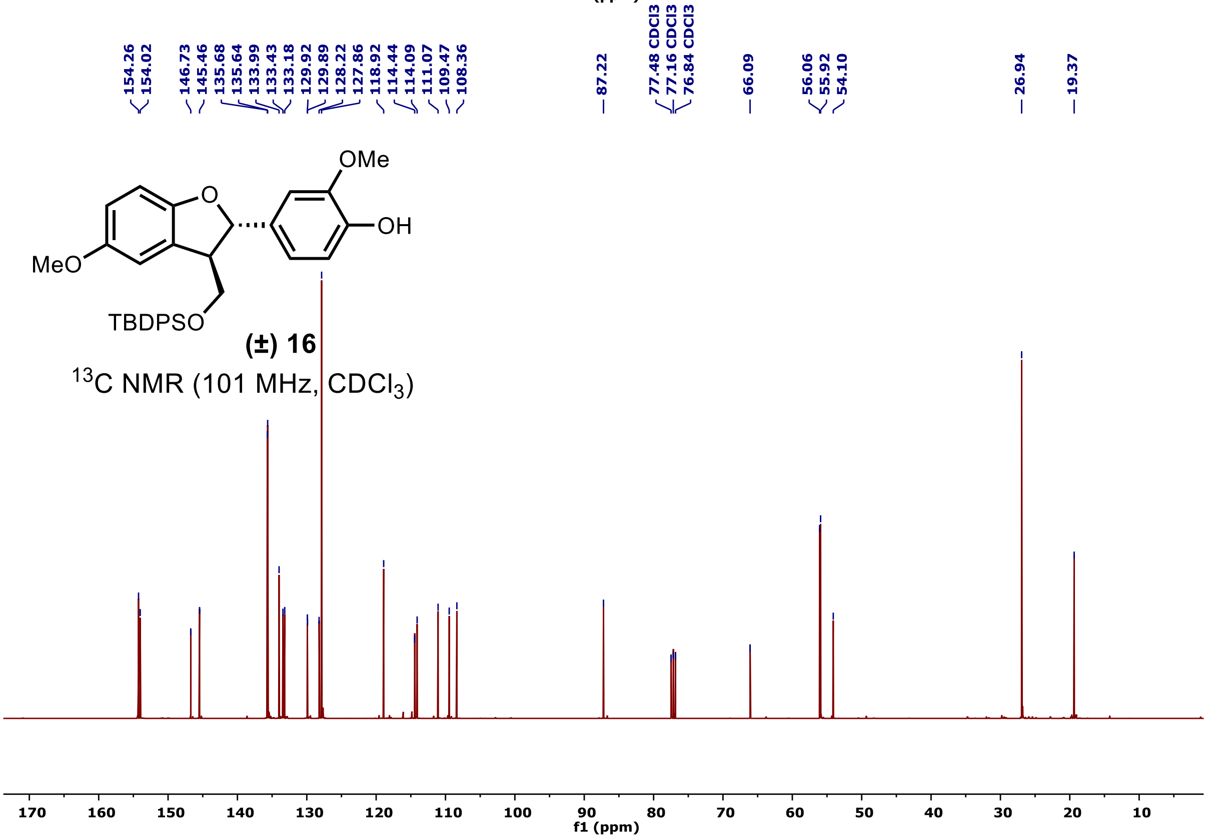
154.26 154.02 146.73 145.46 135.68 135.64 133.99 133.43 133.18 129.92 129.89 128.22 127.86 118.92 114.44 114.09 111.07 109.47 108.36 87.22 77.48 CDCl3 77.16 CDCl3 76.84 CDCl3 66.09 56.06 55.92 54.10 26.94 19.37
OMe
OH
MeO
TBDPSO
(±) 16
13C NMR (101 MHz, CDCl3)
170 160 150 140 130 120 110 100 90 80 70 60 50 40 30 20 10
f1 (ppm)

.

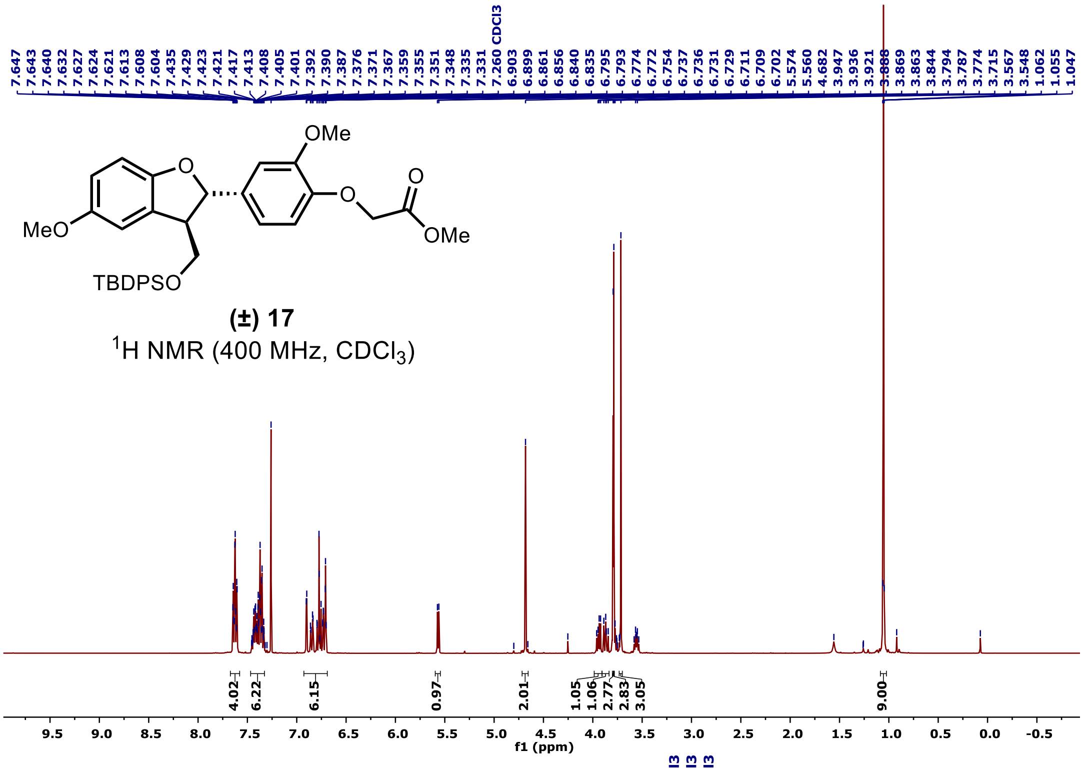
7.647 7.643 7.640 7.632 7.627 7.624 7.621 7.613 7.608 7.604 7.435 7.429 7.423 7.421 7.417 7.413 7.408 7.405 7.401 7.392 7.390 7.387 7.376 7.371 7.367 7.359 7.355 7.351 7.348 7.335 7.331 7.260 CDCl3 6.903 6.899 6.861 6.856 6.840 6.835 6.795 6.793 6.774 6.772 6.754 6.737 6.736 6.731 6.729 6.711 6.709 6.702 5.560 5.574 4.682 3.947 3.936 3.921 3.888 3.869 3.863 3.844 3.794 3.787 3.774 3.715 3.567 3.548 1.062 1.055 1.047
OMe
O
MeO
O
O
OMe
TBDPSO
(±) 17
1H NMR (400 MHz, CDCl3)
4.02
6.22
6.15
0.97
2.01
1.05
1.06
2.77
2.83
3.05
9.00
9.5 9.0 8.5 8.0 7.5 7.0 6.5 6.0 5.5 5.0 4.5 4.0 3.5 3.0 2.5 2.0 1.5 1.0 0.5 0.0 -0.5
f1 (ppm)

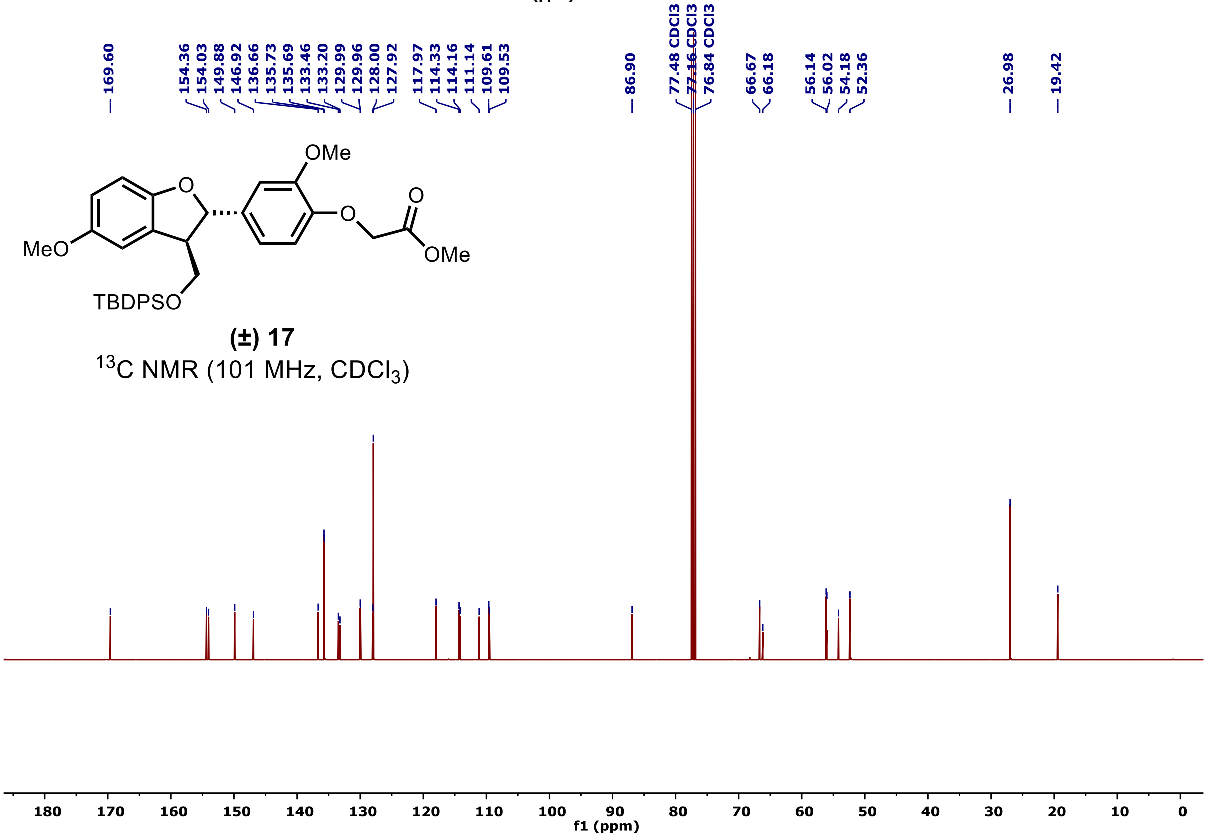
169.60 154.36 154.03 149.88 146.92 136.66 135.73 135.69 133.46 133.20 129.99 129.96 128.00 127.92 117.97 114.33 114.16 111.14 109.61 109.53 86.90 77.48 CDCl3 77.16 CDCl3 76.84 CDCl3 66.67 66.18 56.14 56.02 54.18 52.36 26.98 19.42
OMe
O
MeO
O
O
OMe
TBDPSO
(±) 17
13C NMR (101 MHz, CDCl3)
180 170 160 150 140 130 120 110 100 90 80 70 60 50 40 30 20 10 0
f1 (ppm)

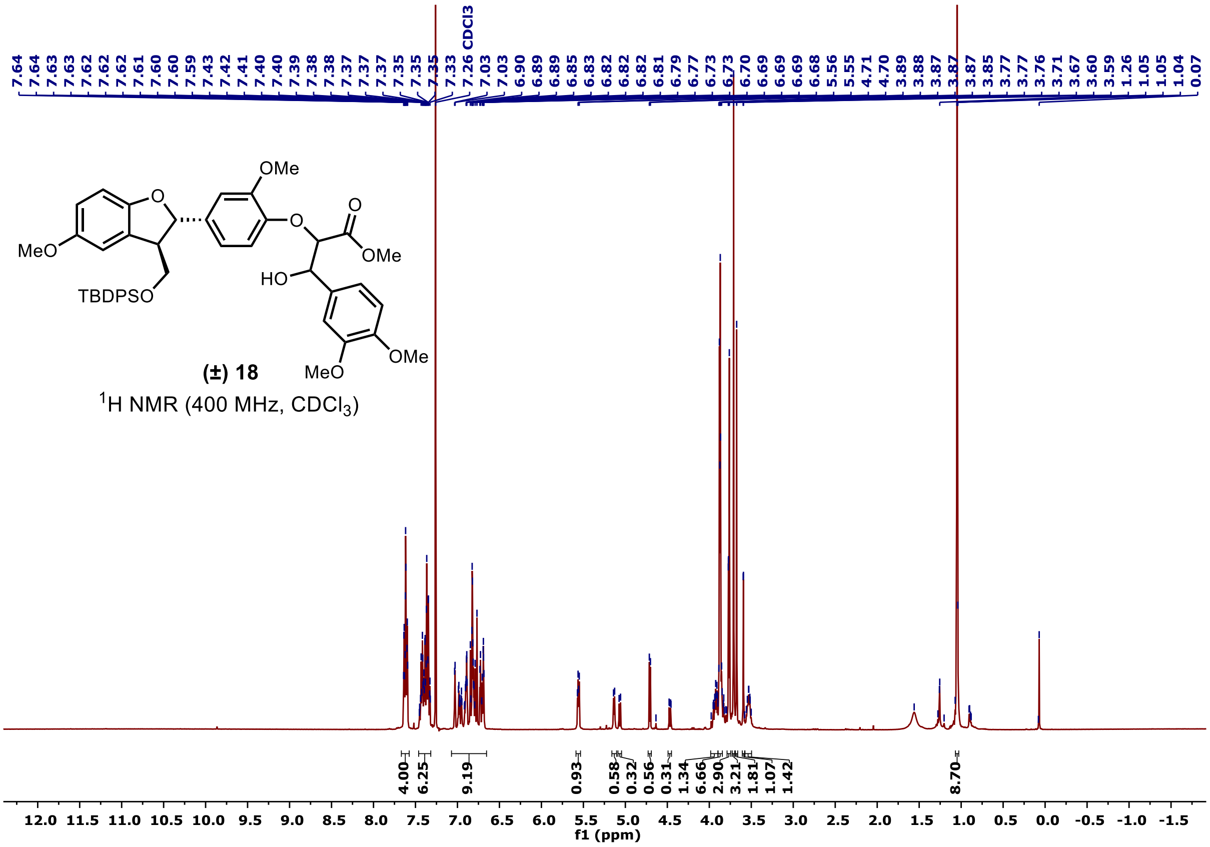
7.64 7.64 7.63 7.62 7.62 7.62 7.61 7.60 7.60 7.59 7.43 7.42 7.41 7.40 7.40 7.39 7.38 7.38 7.37 7.37 7.37 7.35 7.35 7.35 7.33 7.26 CDCl3 7.03 7.03 6.90 6.89 6.89 6.85 6.83 6.82 6.82 6.82 6.81 6.79 6.77 6.73 6.73 6.70 6.69 6.69 6.68 5.56 5.55 4.71 4.70 3.89 3.88 3.87 3.87 3.87 3.85 3.77 3.76 3.71 3.67 3.60 3.59 1.26 1.05 1.04 0.07
OMe
O
O
O
OMe
MeO
TBDPSO
HO
OMe
MeO
(±) 18
^{1}H NMR (400 MHz, $CDCl_3$)
4.00 6.25 9.19 0.93 0.58 0.32 0.56 0.31 1.34 6.66 2.90 3.21 1.81 1.07 1.42 8.70
12.0 11.5 11.0 10.5 10.0 9.5 9.0 8.5 8.0 7.5 7.0 6.5 6.0 5.5 5.0 4.5 4.0 3.5 3.0 2.5 2.0 1.5 1.0 0.5 0.0 -0.5 -1.0 -1.5
f1 (ppm)

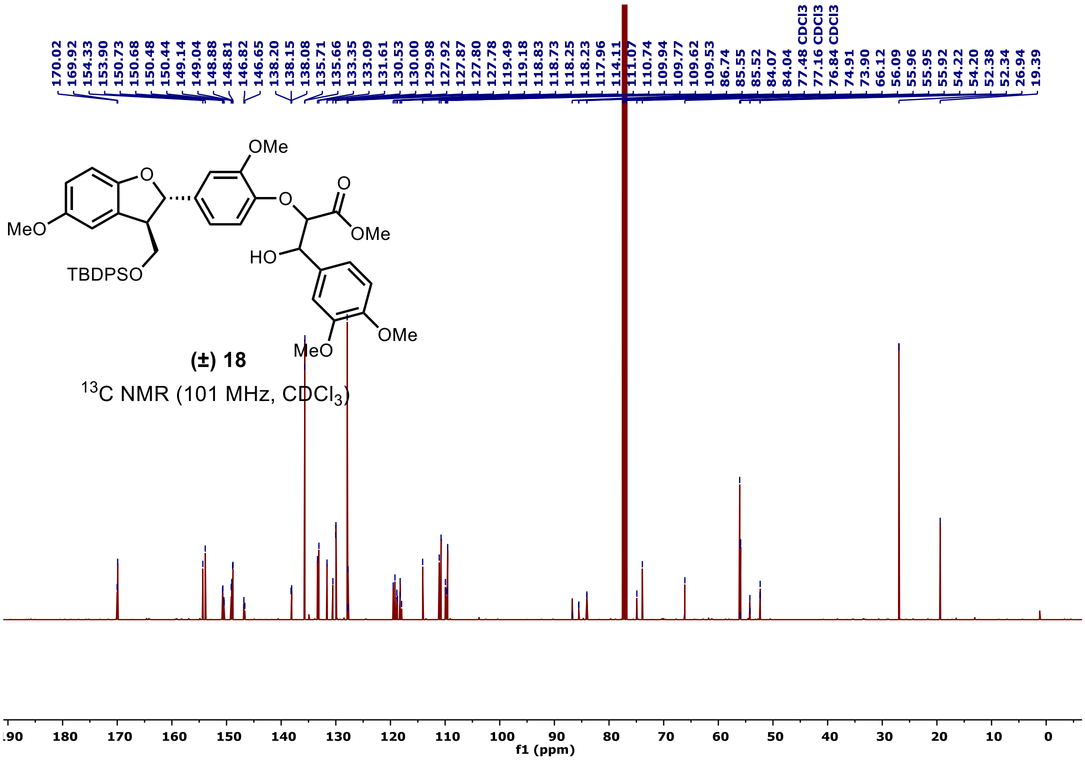
170.02 169.92 154.33 153.90 150.73 150.68 150.48 150.44 149.14 149.04 148.88 148.81 146.82 146.65 138.20 138.15 138.08 135.71 135.66 133.35 133.09 131.61 130.53 130.00 129.98 127.92 127.87 127.80 127.78 119.49 119.18 118.83 118.73 118.25 118.23 117.96 114.11 111.07 110.94 110.74 109.97 109.77 109.62 109.53 86.74 85.55 85.52 84.07 84.04 77.48 CDCl3 77.16 CDCl3 76.84 CDCl3 74.91 73.90 66.12 56.09 55.96 55.95 55.92 54.22 52.38 52.34 26.94 19.39
OMe
O
O
O
OMe
MeO
TBDPSO
HO
OMe
MeO
(±) 18
^{13}C NMR (101 MHz, $CDCl_3$)
190 180 170 160 150 140 130 120 110 100 90 80 70 60 50 40 30 20 10 0
f1 (ppm)

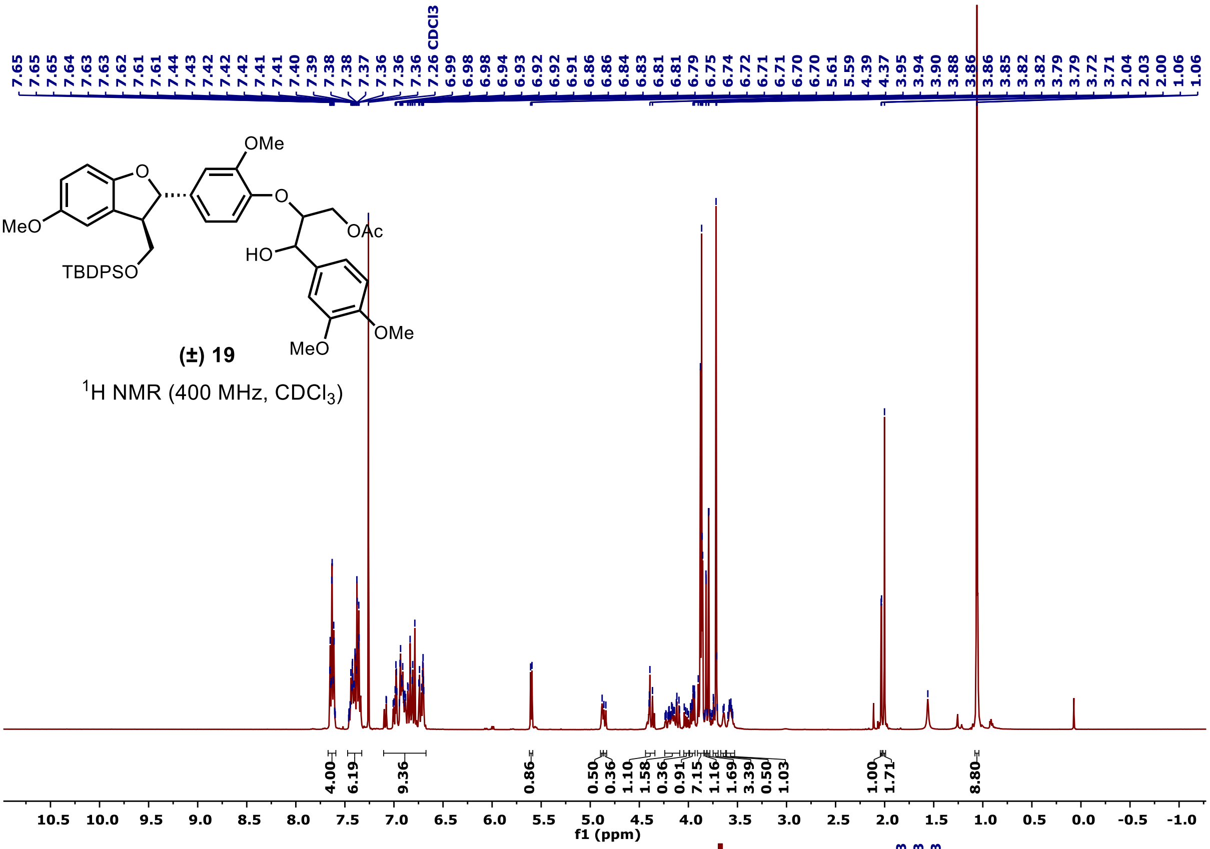
7.65 7.65 7.64 7.63 7.63 7.62 7.61 7.61 7.44 7.43 7.42 7.42 7.41 7.41 7.40 7.39 7.38 7.38 7.37 7.36 7.36 7.26 CDCl3 6.99 6.98 6.98 6.94 6.93 6.92 6.92 6.91 6.86 6.86 6.84 6.83 6.81 6.81 6.79 6.75 6.74 6.72 6.71 6.70 6.70 5.61 5.59 4.39 4.37 3.95 3.94 3.90 3.88 3.86 3.86 3.85 3.82 3.82 3.79 3.79 3.72 3.71 2.04 2.03 2.00 1.06 1.06
OMe
MeO
O
OAc
HO
TBDPSO
MeO
OMe
(±) 19
1H NMR (400 MHz, CDCl3)
4.00 6.19 9.36 0.86 0.50 0.36 1.10 1.58 0.36 0.91 7.15 1.16 1.69 3.39 0.50 1.03 1.00 1.71 8.80
f1 (ppm)

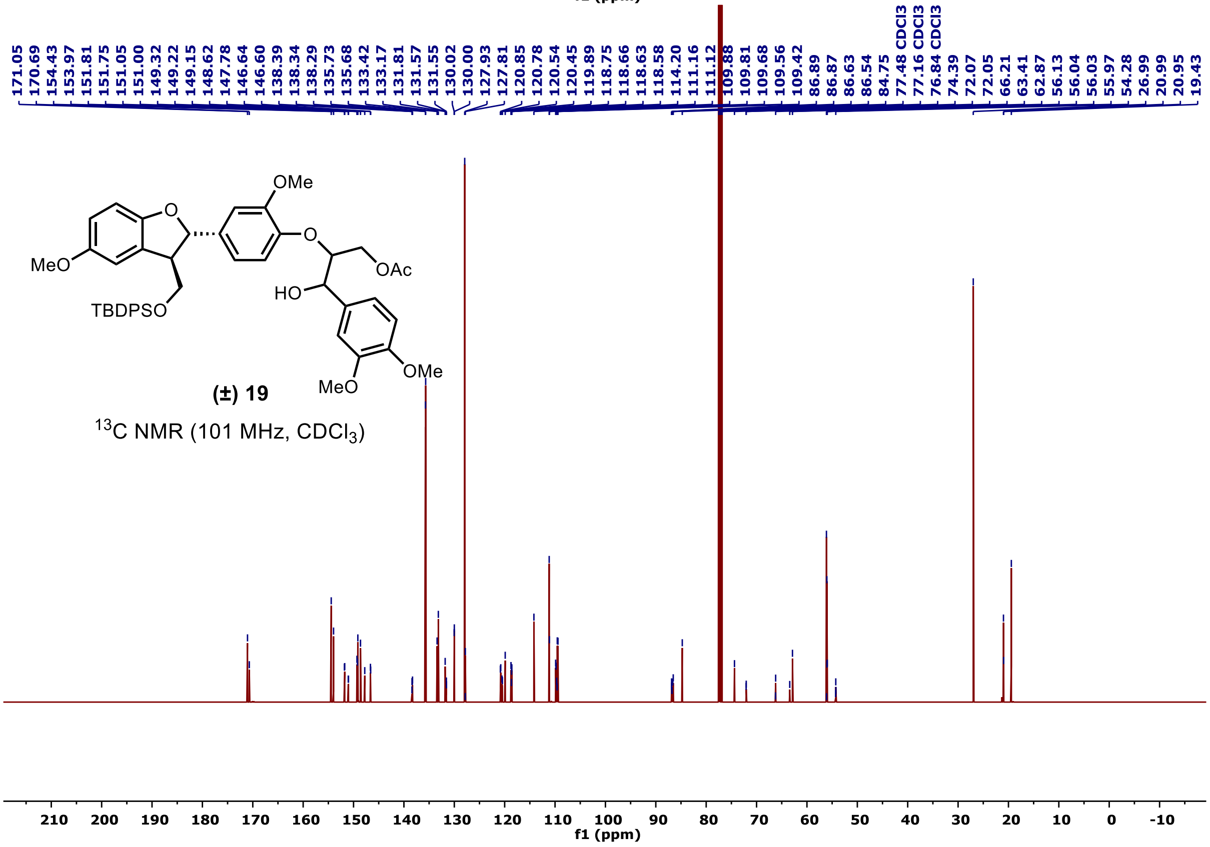
171.05 170.69 154.43 153.97 151.81 151.75 151.05 151.00 149.32 149.22 149.15 148.62 147.78 146.64 146.60 138.39 138.34 138.29 135.73 135.68 133.42 133.17 131.81 131.57 131.55 130.02 130.00 127.93 127.81 120.85 120.78 120.54 120.45 119.89 118.75 118.66 118.63 118.58 114.20 111.16 111.12 109.88 109.81 109.68 109.56 109.42 86.89 86.87 86.63 86.54 84.75 77.48 CDCl3 77.16 CDCl3 76.84 CDCl3 74.39 72.07 72.05 66.21 63.41 62.87 56.13 56.04 56.03 55.97 54.28 26.99 20.99 20.95 19.43
OMe
MeO
O
OAc
HO
TBDPSO
MeO
OMe
(±) 19
13C NMR (101 MHz, CDCl3)
f1 (ppm)

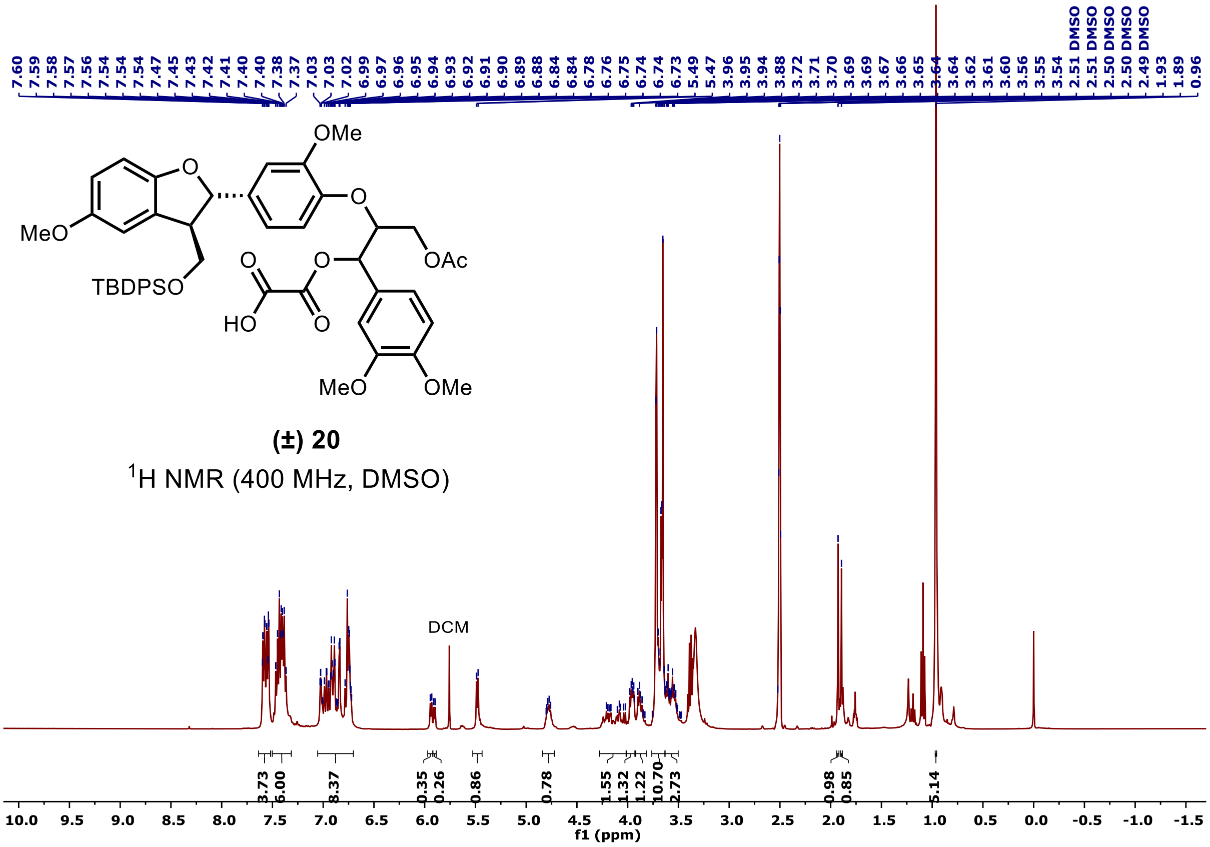
7.60 7.59 7.58 7.57 7.56 7.54 7.54 7.54 7.47 7.45 7.43 7.42 7.41 7.40 7.40 7.38 7.37 7.03 7.03 7.02 6.99 6.97 6.96 6.95 6.94 6.93 6.92 6.91 6.90 6.89 6.88 6.84 6.84 6.78 6.76 6.75 6.74 6.74 6.73 5.49 5.47 3.96 3.95 3.94 3.88 3.72 3.71 3.70 3.69 3.69 3.67 3.66 3.65 3.64 3.64 3.62 3.61 3.60 3.56 3.55 3.54 2.51 DMSO 2.51 DMSO 2.50 DMSO 2.50 DMSO 2.49 DMSO 1.93 1.89 0.96
OMe
O
MeO
TBDPSO
O
OAc
O
HO
O
MeO
OMe
(±) 20
1H NMR (400 MHz, DMSO)
DCM
3.73 6.00 3.37 0.35 0.26 0.86 0.78 1.55 1.32 1.22 10.70 2.73 0.98 0.85 5.14
10.0 9.5 9.0 8.5 8.0 7.5 7.0 6.5 6.0 5.5 5.0 4.5 4.0 3.5 3.0 2.5 2.0 1.5 1.0 0.5 0.0 -0.5 -1.0 -1.5
f1 (ppm)

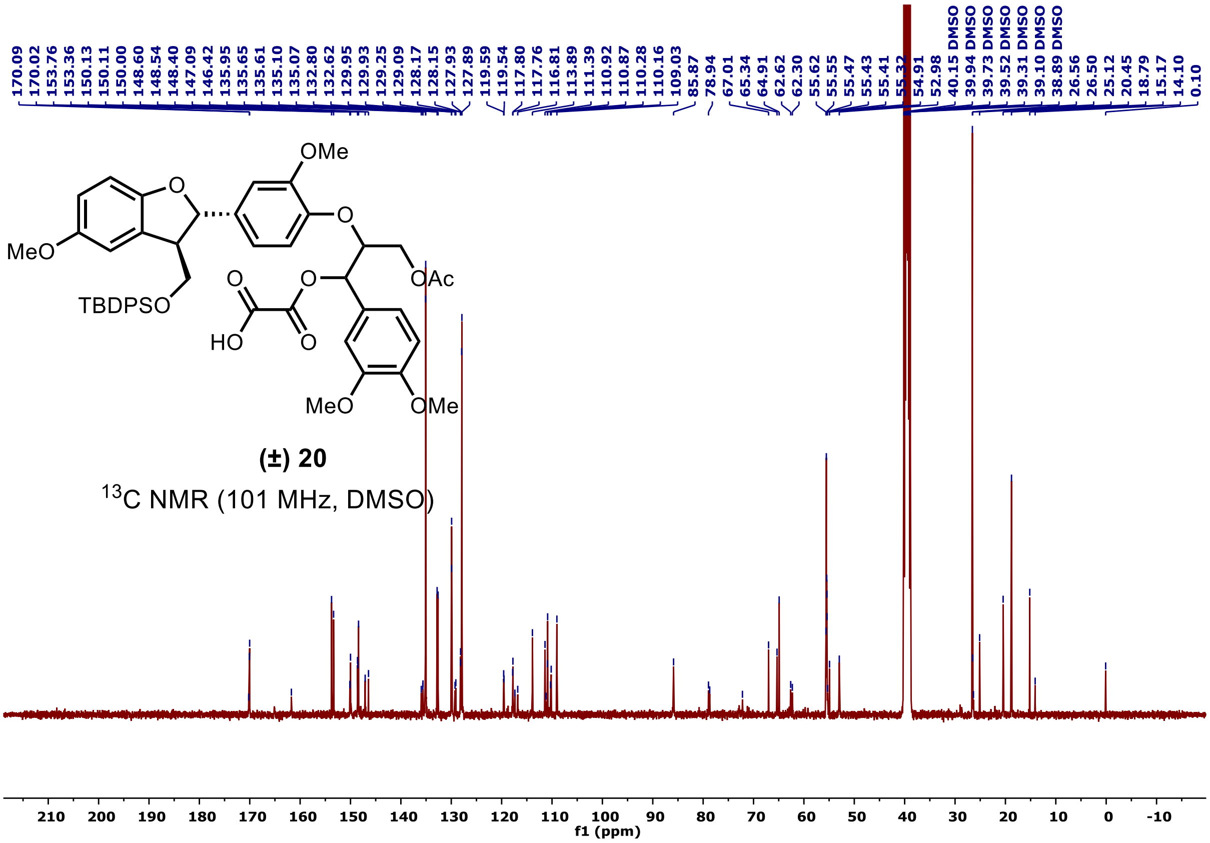
170.09 170.02 153.76 153.36 150.13 150.11 150.00 148.60 148.54 148.40 147.09 146.42 135.95 135.65 135.61 135.10 135.07 132.80 132.62 129.95 129.93 129.25 129.09 128.17 128.15 127.93 127.89 119.59 119.54 117.80 117.76 116.81 113.89 111.39 110.92 110.87 110.28 110.16 109.03 85.87 78.94 67.01 65.34 64.91 62.62 62.30 55.62 55.55 55.47 55.43 55.41 55.32 54.91 52.98 40.15 DMSO 39.94 DMSO 39.73 DMSO 39.52 DMSO 39.31 DMSO 39.10 DMSO 38.89 DMSO 26.56 26.50 25.12 20.45 18.79 15.17 14.10 0.10
OMe
O
MeO
TBDPSO
O
OAc
O
HO
O
MeO
OMe
(±) 20
13C NMR (101 MHz, DMSO)
210 200 190 180 170 160 150 140 130 120 110 100 90 80 70 60 50 40 30 20 10 0 -10
f1 (ppm)

7. References

1) Choi, G. B.; Zhu, Q.; Miller, D. C.; Gu, C. J.; Knowles, R. R. Catalytic alkylation of remote C–H bonds enabled by proton-coupled electron transfer. *Nature*, **2016**, *539*, 268.
2) Kärkäs, M. D.; Bosque, I.; Matsuura, B. S.; Stephenson, C. R. J. Photocatalytic Oxidation of Lignin Model Systems by Merging Visible-light Photoredox and Palladium Catalysis. *Org. Lett.* **2016**, *18*, 5166–5169.
3) Bomon, J.; Van Den Broeck, E.; Bal, M.; Liao, Y.; Sergeyev, S.; Van Speybroeck, V.; Sels, B. F.; Maes, B. U. W. Brønsted Acid Catalyzed Tandem Defunctionalization of Biorenewable Ferulic acid and Derivates into Bio-Catechol. *Angew. Chem. Int. Ed.* **2020**, *59*, 3063–3068.
4) Nguyen, J. D.; Matsuura, B. S.; Stephenson, C. R. J. A Photochemical Strategy for Lignin Degradation at Room Temperature. *J. Am. Chem. Soc.* **2014**, *136*, 1218–1221.
5) Zhang, Q.; Kang, X.; Long, L; Zhu, L.; Chai, Y. Mild and Selective Deprotection of tert-butyl(dimethyl)silyl Ethers with Catalytic Amounts of Sodium Tetrachloroaurate(III) Dihydrate. *Synthesis*, **2015**, *47*, 55-64.
6) Campbell, A. N.; White, P. B.; Guzei, I. A.; Stahl, S. S. Allylic C−H Acetoxylation with a 4,5-Diazafluorenone-Ligated Palladium Catalyst: A Ligand-Based Strategy To Achieve Aerobic Catalytic Turnover. *J. Am. Chem. Soc.* **2010**, *132*, 15116–15119.
